# Effect of the Thermal Activation on the Adsorption Capacity of Cationic and Anionic Dyes in Magnetic Carbon

**DOI:** 10.1021/acsomega.5c05783

**Published:** 2025-08-05

**Authors:** Gabriela A. Nogueira, Jaqueline R. Lopes, Eduardo C. Vilas Boas, Thiago N. M. Cervantes, Altair B. Moreira, Odair P. Ferreira, Márcia C. Bisinoti

**Affiliations:** † Department of Chemistry and Environmental Sciences (DQCA), Laboratory of Environmental Sciences Studies (LECA), São Paulo State University (UNESP), Institute of Biosciences, Letters and Exact Sciences (IBILCE), 2265 Cristóvão Colombo Street, Jardim Nazareth, São José Do Rio Preto − SP, ZIP code, São José 15054-000, Brazil; ‡ Department of Chemistry, State University of Londrina (UEL), Advanced Functional Materials Laboratory (LaMFA), Celso Garcia Cid Highway, PR 445, km 380, Londrina − PR, ZIP code 86055-900, Brazil; § Department of Chemistry, State University of Londrina (UEL), Laboratory of Environmental Electrochemistry (LabEA), Celso Garcia Cid Highway, PR 445, km 380, Londrina − PR, ZIP code 86055-900, Brazil

## Abstract

The sustainable synthesis of multifunctional magnetic carbons was achieved using sugar cane bagasse by hydrothermal carbonization with ferric nitrate, followed by thermal activation under CO_2_ and N_2_ at 500–900 °C. Structural, magnetic, and surface characterizations were performed to evaluate their physicochemical properties and explore their potential for environmental applications, including the adsorption of cationic and anionic dyes. Activation at 700 °C significantly enhanced the material properties, particularly under N_2_, yielding a high specific surface area (241 m^2^ g^–1^), notable magnetization (27.4 emu g^–1^), and a low *I*_D_/*I*_G_ ratio (0.42), indicative of graphitic domains. While CO_2_ activation led predominantly to magnetite formation, N_2_ favored the formation of iron carbide and zero-valent iron. The materials exhibited high adsorption capacities for methylene blue (MB; 81.4 mg g^–1^) and reactive blue 19 (RB19; 74.8 mg g^–1^). Adsorption kinetics followed mixed mechanisms involving both physisorption and chemisorption, while the Sips isotherm model best described the equilibrium, suggesting heterogeneous surface interactions. Activation at 700 °C under N_2_ was particularly effective, enhancing MB and RB19 adsorption by up to 5.5- and 15.5-fold, respectively. This performance was mainly attributed to the increased specific surface area and pore volume, which facilitate dye diffusion and retention. The N_2_ atmosphere limited carbon oxidation, promoting the development of mesoporous structures that efficiently adsorb both cationic and anionic dyes, underscoring the multifunctionality and sustainability of these materials for environmental applications.

## Graphical abstract

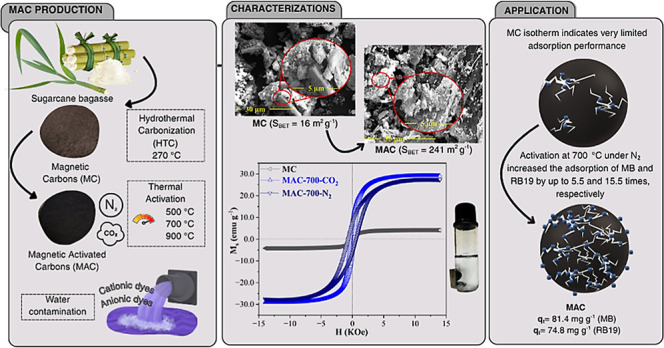

## 1 Introduction

Dyes are widely used in the textile, rubber, cosmetics, and leather industries, leading to the discharge of colored effluents that pose environmental and ecological risks due to their chemical stability and toxicity.^1,2^ Among them, methylene blue (MB) and Reactive Blue 19 (RB19) are frequently used cationic and anionic dyes, respectively.^3,4^ These dyes are recalcitrant due to their complex chemical structures and low reactivity, making them resistant to degradation. Additionally, they can negatively affect the photic zone and hinder the growth of aquatic organisms, with many exhibiting toxic properties.^5^

Although several methods have been employed for dye removal, such as adsorption, ion exchange, membrane filtration, coagulation–flocculation, and advanced oxidation processes,^3^ adsorption has emerged as a particularly effective and sustainable approach due to its simplicity and cost-effectiveness.^3,4^ Adsorbents with high surface area, such as alumina, activated carbon, zeolite, and silica gel, are widely used in adsorption processes.^3,6–8^ Magnetic carbon (MC) has recently gained attention due to its high chemical and thermal stability, surface versatility, use of biomass as a precursor, and magnetic properties that facilitate more efficient solid–liquid separation.^9^

Recent studies have explored the feasibility of using agricultural residues, such as sugar cane bagasse^10–12^ and green pea shells,^13,14^ as precursors to produce magnetic carbon (MC), which are carbonaceous materials embedded with magnetic nanoparticles.^9,10,15,16^ These materials are primarily synthesized through pyrolysis or hydrothermal carbonization (HTC),^15,17,18^ with HTC considered more efficient due to the elimination of the predrying step following the mixing with magnetic precursors.^10^ In HTC, the biomass is suspended in water and heated in a sealed system at 175–300 °C under autogenous pressure.^15,19^ This process imparts magnetism at relatively low temperatures, thereby reducing energy consumption.^15,20^

The MC produced by HTC exhibits low porosity and specific surface area, resulting in poor adsorption efficiency.^10,11,16^ To enhance its properties, chemical, physical, or physicochemical activations are required.^17,21,22^ In chemical activation, agents such as KOH, H_3_PO_4_, or ZnCl_2_ are incorporated into the carbonaceous material.^23–25^ In physicochemical activation, this is followed by thermal treatment at moderate temperatures and subsequent washing steps to remove excess activating agents, which may generate residual waste.^17,21,26,27^ In contrast, physical activation, such as high-temperature thermal treatment (700–1000 °C) in steam, inert, or oxidizing atmospheres, eliminates the need for post-treatment washing, resulting in minimal waste generation.^28–30^ These techniques not only increase the material’s porosity and specific surface area but also modify pore size distribution, which directly affects its adsorption performance.^17,23^ However, many studies emphasize adsorption capacity without exploring the fundamental relationships between synthesis and activation conditions, such as temperature variation and gaseous atmospheres, and the intrinsic characteristics of materials with their responses as adsorbents, which ensures the possibility of multifunctional use of these materials.

The production of magnetic activated carbons (MACs) through the thermal activation of MC in N_2_ or CO_2_ atmospheres shows potential for reducing waste generation during activation. This approach can also promote a favorable combination of magnetic properties and high porosity, which can result in greater adsorption capacity for dyes such as MB and RB19. In this context, the present study aims to elucidate the influence of CO_2_ and N_2_ atmospheres at 500, 700, and 900 °C on the properties of MACs derived from sugar cane bagasse. The goal is not only to enhance adsorption performance for MB and RB19 but also to provide insights into the structure–function relationship driven by activation parameters. Such comparative studies are scarce in the literature, despite the widespread use of MCs. This work contributes to the rational design of sustainable materials for wastewater treatment based on renewable precursors and activation protocols with reduced environmental impact.

## 2 Results and Discussion

### 2.1 Characterizations

#### 2.1.1 Compositional and Structural Analysis

After the HTC of sugar cane bagasse in the presence of ferric nitrate, an increase in carbon content (∼22%) and a reduction in hydrogen and nitrogen contents (∼50% and 69%, respectively) were observed compared to the raw bagasse (Table 1). This behavior is attributed to the reactions promoted during the HTC process, such as dehydration, decarboxylation, and polymerization, which enhance carbon content while reducing hydrogen and nitrogen levels.^31^

**Table 1:** CHN elemental analysis and contents of iron, adsorbed water, and inorganic residue

Samples	Elemental composition (%)			Estimated iron content (%)^a^	Adsorbed water (%)^b^	Inorganic residue (%)^c^
	C	H	N			
Sugar cane	46.5	6.6	5.2	-	2.5	1.5^d^
**MC**	56.8	3.3	1.6	20.2	2.5	28.2
**MAC-500-CO**_**2**_	57.9	3.0	2.0	25.7	4.1	36.0
**MAC-700-CO**_**2**_	58.6	2.0	2.1	35.0	2.3	50.2
**MAC-900-CO**_**2**_	1.0	-	0.1	71.5	0.2	102.0
**MAC-500-N**_**2**_	59.8	2.6	1.9	27.6	3.4	38.9
**MAC-700-N**_**2**_	59.9	2.0	1.1	36.8	2.0	51.4
**MAC-900-N**_**2**_	57.3	-	1.6	38.1	1.2	53.7

a Calculated using TGA residue as Fe_2_O_3_ using eqs S8 and S9.

b Measured as a percentage of mass loss <130 °C.

c Residue value of the TGA curve at 700 °C.

d Ash is mainly composed of SiO_2_.^11,32^ - Not detected.

MC activation at 500 °C under a CO_2_ atmosphere (MAC-500-CO_2_) did not lead to significant changes in the material’s composition (Table 1). However, activation under a N_2_ atmosphere resulted in a 5% increase in carbon content. A similar trend was observed when the activation temperature of MC was raised to 700 °C under both CO_2_ and N_2_ atmospheres. Activation at 900 °C under CO_2_ led to a drastic reduction in the concentrations of carbon, hydrogen, and nitrogen (∼98%, 100%, and 94%, respectively). This sample exhibited a high iron content (∼72%), indicating that nearly all the carbonaceous matrix had been removed. In contrast, under N_2_ atmosphere, complete removal of hydrogen (100%) was observed, which at high temperatures is released as volatile compounds (H_2_, H_2_O, and light hydrocarbons).^33,34^ Activation under N_2_ favors the preservation of carbon in the material, as the inert nature of the gas minimizes losses due to oxidation or degradation, which are more pronounced under CO_2_.^34,35^ Additionally, the removal of surface oxygen-containing functional groups (e.g., carboxyl or hydroxyl groups) may contribute to the relative increase in carbon content.^33–35^

TG/DTG curves for sugar cane bagasse revealed four distinct thermal events (Figure S1). The first event, occurring between 30 and 130 °C, was attributed to the loss of adsorbed water (Table 1). The second event, identified at 325 °C, corresponded to the degradation of hemicellulose. Cellulose decomposition was observed during the third event, recorded at 365 °C. The final thermal event, detected at 480 °C, was associated with the degradation of lignin, in agreement with previously reported findings in the literature.^18,36,37^

After HTC, three distinct thermal events were identified in the TG curve (Figure S1). For the MC, the observed events corresponded to the loss of adsorbed water, the decomposition of residual organic constituents from sugar cane bagasse, including amorphous carbon fractions, and the release of volatile compounds during the oxidation of iron species present in the material.^19,38^ Thermal activation at 500 °C under both atmospheres reduced the number of thermal events from three to two (Figure S1). The first event, observed at approximately 130 °C, was associated with the loss of surface-adsorbed water (Table 1). The second thermal event, occurring in the range of 300 to 700 °C, was attributed to the degradation of polycondensed aromatic structures and the more stable organic fractions.^10,38^ With the increase in activation temperature to 700 °C under CO_2_ and N_2_ atmospheres, a gradual rise in the onset temperature of thermal decomposition was observed, indicating enhanced thermal stability of the materials activated at higher temperatures. This behavior reflects the greater thermal resistance of the activated materials, possibly due to the formation of more ordered and compact carbonaceous structures.

Following the HTC process, an increase in the TG residue was observed (Table S1), suggesting that the hydrothermal decomposition of sugar cane bagasse in the presence of ferric nitrate promoted the incorporation of iron into the samples (Table 1). As the activation temperature increased, under both CO_2_ and N_2_ atmospheres, the iron content in the samples also increased (Table 1). This trend can be attributed to the removal of organic and carbonaceous fractions, which facilitates the concentration of iron in the remaining material. This is consistent with the thermal decomposition of organic matrices, which occurs more intensely at elevated temperatures, allowing for the retention and potential redistribution of metal species in the activated material.^35,39^ In the MAC-900-CO_2_, the TG residue exceeded 100%, which can be attributed to sample oxidation during heating, as it is composed predominantly of inorganic material, resulting in a mass gain due to the formation of oxides (Table 1).

XRD pattern obtained for sugar cane bagasse (Figure S2) exhibits broad peaks at 16.8° and 22.1° (2θ), indicating the presence of crystalline cellulose (ICDD: 00-050-2241), superimposed on the characteristic halo of a poorly ordered solid, attributed to the other constituents of the biomass.^10,40^ In contrast, for the MC (Figure S2), the amorphous halo remains, while the peak at 16.8°, associated with crystalline cellulose, is no longer observed. This behavior can be attributed to the partial degradation of cellulose under hydrothermal conditions, which typically begins at approximately 230 °C.^31,41,42^ Following activation under CO_2_ and N_2_ atmospheres, the characteristic halo was still observed. However, its intensity was reduced when compared to that of the inorganic phases present in the samples (Figure 1a,b).

**Figure 1:**
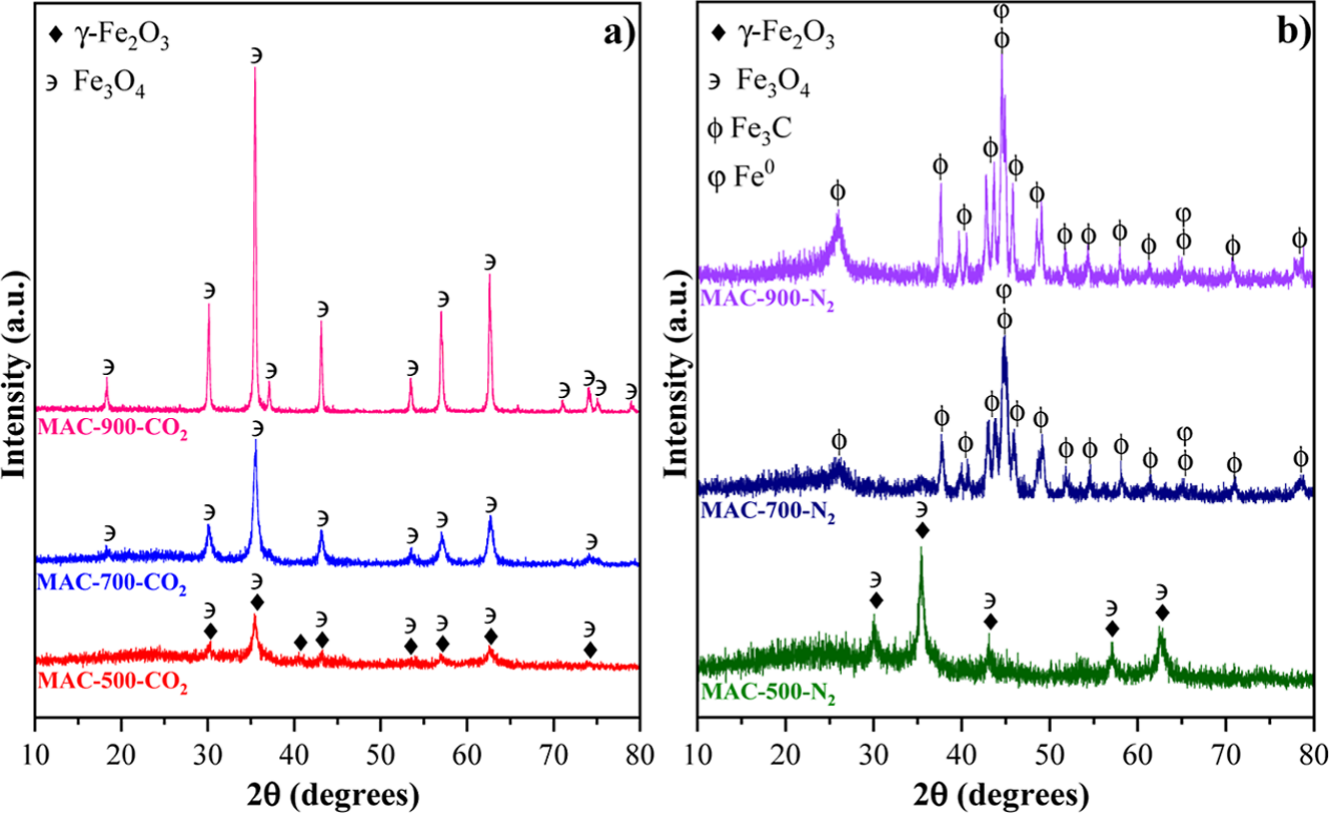
XRD patterns corresponding to the samples (a) MAC-500-CO_2_, MAC-700-CO_2_, and MAC-900-CO_2_; and (b) MAC-500-N_2_, MAC-700-N_2_, and MAC-900-N_2_.

After the HTC process in the presence of ferric nitrate, the hematite phase (α-Fe_2_O_3_–non-magnetic), characterized by a rhombohedral crystal system and space group R3̅c (ICDD: 01-073-0603), was identified by diffraction peaks at 33.2°, 35.6°, 40.3°, 57.2°, and 62.8° (2θ) (Figure S2). Additionally, the maghemite phase (γ-Fe_2_O_3_–magnetic), which exhibits a cubic crystal system and space group *P*4_1_32 (ICDD: 00-039-1346), was also detected, with characteristic peaks at 26.6°, 30.4°, 33.2°, 35.6°, 40.3°, 54.0°, 57.2°, and 62.6° (2θ). Peaks at 35.6°, 54.0°, 57.2°, and 62.6° (2θ) also suggest the presence of magnetite (Fe_3_O_4_–magnetic), which possesses an orthorhombic crystal system and space group *Imma* (ICDD: 01-075-1609).

Activation at 500 °C under CO_2_ and N_2_ atmospheres eliminated the hematite phase, with only maghemite and magnetite remaining (Figure 1a,b). When the activation temperature increased to 700 °C, the magnetite phase was preserved in the sample activated under a CO_2_ atmosphere (Figure 1a). However, for activation under a N_2_ atmosphere, the formation of iron carbide (Fe_3_C–magnetic) was detected. This phase exhibits an orthorhombic crystal structure and belongs to the Pbnm space group (ICDD: 01-075-0910), as evidenced by diffraction peaks located at 26.2°, 37.8°, 39.9°, 40.7°, 43.0°, 43.8°, 44.7°, 45.1°, 45.9°, 49.2°, 51.8°, 54.5°, 58.2°, 61.5°, 65.1°, 71.0°, and 78.4° (2θ) (Figure 1b). Additionally, peak overlap at 44.7° and 65.1° (2θ) suggests the presence of the zero-valent iron phase (Fe^0^–magnetic; ICDD: 01-087-0721). The identified phases are consistent with the elemental mapping of iron, carbon, and oxygen in these samples (Figure S3). The MAC-700-N_2_ sample showed no surface oxygen, unlike MAC-700-CO_2_, confirming that Fe_3_C and Fe^0^ are indeed the predominant phases in MAC-700-N_2_. A similar behavior was observed during activation at 900 °C, where the magnetite phase remained in the material activated under CO_2_, while iron carbide and zero-valent iron phases were present in the sample activated under N_2_ (Figure 1a,b). Equations 1–5 describe the formation reactions of the magnetite, iron carbide, and zero-valent iron phases, by the mechanisms proposed by Lv et al.^43^ and Vieira et al.^16^

1 $$3{Fe}_{2}{O_{3}}_{\left(s\right)}+{\left(H_{2},C,CO,C_{x}H_{y}\right)}_{\left(g\right)}\to 2{Fe}_{3}{O_{4}}_{\left(s\right)}+{\left(H_{2}O,CO,{CO}_{2},C_{x}H_{y}O_{z}\right)}_{\left(g\right)}$$

2 $${Fe}_{3}{O_{4}}_{\left(s\right)}+{3C}_{\left(s\right)}\to {{Fe}_{3}C}_{\left(s\right)}+{2{CO}_{2}}_{\left(g\right)}$$

3 $$6{Fe}_{2}{O_{3}}_{\left(s\right)}+C_{\left(s\right)}\to 4{Fe}_{3}{O_{4}}_{\left(s\right)}+{CO_{2}}_{\left(g\right)}$$

4 $${Fe}_{3}{O_{4}}_{\left(s\right)}+C_{\left(s\right)}\to 3{FeO}_{\left(s\right)}+{CO}_{\left(g\right)}$$

5 $${FeO}_{\left(s\right)}+{CO}_{\left(g\right)}\to {Fe}_{\left(s\right)}+{{CO}_{2}}_{\left(g\right)}$$

This behavior can be attributed to the distinct interactions between the activating gases and the precursor material. Under a CO_2_ atmosphere, even at elevated temperatures, the gas selectively oxidizes the material’s surface, promoting the formation of iron oxides such as magnetite.^44^ In contrast, under an inert N_2_ atmosphere, increasing the temperature to 700 and 900 °C creates favorable conditions for the partial reduction of iron oxides and their subsequent reaction with the carbon in the material, leading to the formation of iron carbides and zero-valent iron.^45^

FTIR analysis of the sugar cane bagasse (Figure S4) shows a broad and intense band in the 3400–3000 cm^–1^, centered at 3343 cm^–1^, attributed to O–H stretching vibrations, characteristic of hydroxyl groups. Bands at 2964, 2903, and 2851 cm^–1^ are associated with asymmetric and symmetric aliphatic C–H bonds stretching from CH_3_, CH_2_, and CH groups. The band at 1726 cm^–1^ corresponds to C=O stretching vibrations from COOR or COOH groups.^46,47^ Additionally, bands at 1601 and 1505 cm^–1^ are assigned to C=C stretching vibrations of aromatic rings.^48,49^ Bands at 1441 and 1377 cm^–1^ are attributed to C–H bending vibrations, while the characteristic C–O–C stretching band of cellulose chains appears at 1248 cm^–1^. In the 1149–835 cm^–1^ range, bands corresponding to stretching and bending vibrations of C–O–C, C–O, and C–OH groups are observed.^11,32^

After HTC, the MC (Figure S4) exhibited a reduction in the intensity of carbonaceous bands seen in the FTIR spectrum of the raw bagasse. Subsequent activation at 500 °C under CO_2_ and N_2_ atmospheres (Figure 2a,b) led to a marked decrease in the intensities of the bands at 3343, 1726, 1505, 1441, 1377, and 1248 cm^–1^, related to the vibrational modes of O–H, C=O, C=C, C–H, and C–O–C bonds, respectively. This reduction is attributed to dehydration and deoxygenation reactions occurring during MC activation processes.^10,23,29,30^

**Figure 2:**
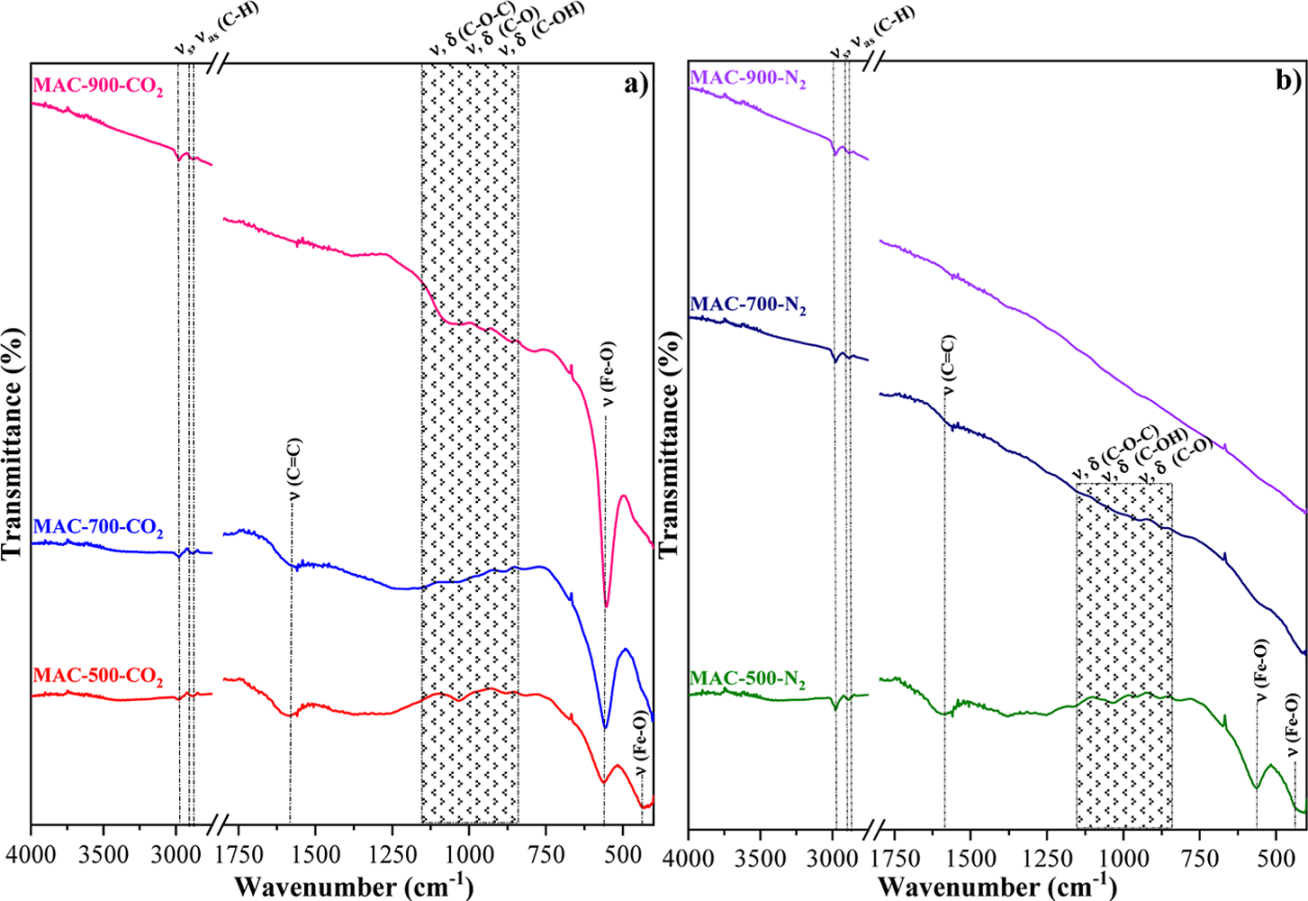
FTIR spectra of the samples (a) MAC-500-CO_2_, MAC-700-CO_2_, and MAC-900-CO_2_; and (b) MAC-500-N_2_, MAC-700-N_2_, and MAC-900-N_2_.

Activation at 700 °C (Figure 2a,b) under both CO_2_ and N_2_ atmospheres resulted in behavior similar to that observed for carbonaceous phases at 500 °C. However, at 900 °C, activation under CO_2_ (Figure 2a) resulted in a spectral profile comparable to that at lower temperatures, whereas activation under N_2_ (Figure 2b) led to a reduction in the intensity of the bands at 1601, 1149, and 835 cm^–1^, suggesting increased symmetry in the graphitic domains, which become undetectable by infrared spectroscopy.

Bands centered at 564 and 439 cm^–1^ in samples MC, MAC-500-CO_2_, and MAC-500-N_2_ indicate the presence of iron oxides (Fe–O), such as hematite, maghemite, and magnetite, corroborating XRD results (Figures S1, 1a,b). Activation at 700 °C under CO_2_ led to a marked decrease in the 439 cm^–1^ band, indicating partial conversion of iron oxides into other iron phases. This behavior aligns with XRD data showing predominant magnetite in this sample (Figure 1a). In contrast, activation at 700 °C under N_2_ did not display Fe–O characteristic bands, suggesting the formation of other iron phases, such as iron carbide and zero=valent iron, as confirmed by XRD (Figure 1b). A similar trend was observed at 900 °C, with Fe–O bands detected in MAC-900-CO_2_ but absent in MAC-900-N_2_ (Figure 2a,b).

Raman spectra in the range of 250–3000 cm^–1^ for the MC and those activated under CO_2_ or N_2_ atmospheres at 500, 700, and 900 °C (Figure S5) displayed two characteristic bands of carbonaceous materials at approximately 1365 and 1600 cm^–1^, corresponding to the D and G bands, respectively. The D band is associated with disordered regions, typically linked to defects, vacancies, heteroatom incorporation (e.g., nitrogen), and sp^3^-hybridized carbon, while the G band corresponds to ordered graphitic structures (sp^2^ domains).^16^

MC spectrum (Figure S5a) exhibited a broad band between 1750 and 2100 cm^–1^, attributed to fluorescence. The intensity of this fluorescence decreased after thermal treatment under CO_2_ and N_2_ atmospheres (Figure S5b,c). As fluorescence has a higher quantum yield than Raman scattering, it is commonly observed in π-electron systems such as MC.^50^ High fluorescence intensity is also linked to amorphous components, which are prominent in samples treated at 500 °C under both atmospheres (Figure S5b,c). Thus, the fluorescence reduction observed after thermal treatment suggests increased structural order compared to the MC, in agreement with the decreased full width at half-maximum and increased peak intensity in the XRD patterns of MACs (Figure 1) relative to MC (Figure S2).

Carbonaceous phase in the nanocomposites was further examined by analyzing the intensity ratio of the D and G bands (*I*_D_/*I*_G_).^51^ To this end, the spectra in Figure S4a–S4c were deconvoluted in the 1150–1750 cm^–1^ region using the Voigt function,^52^ enabling calculation of the *I*_D_/*I*_G_ ratio and identification of sub-bands: D_1_ and D_2_ within the D band, and G^–^ and G^+^ within the G band.

Deconvoluted spectra (Figure S5d–j) revealed four vibrational modes: a minor peak at ∼1235 cm^–1^ attributed to aryl–OH stretching,^53^ likely a remnant of the original biomass (sugar cane bagasse), which is absent in the samples treated at 900 °C; the D_1_ band (∼1365 cm^–1^); the G^–^ mode (∼1560 cm^–1^); and the G^+^ mode (∼1600 cm^–1^). While the D band remained symmetrical, the G band split into G^–^ and G^+^, a feature commonly observed in mesoporous carbon materials such as MACs, and sensitive to temperature variations.^54^ This splitting is an indicator for assessing the thermal effects on the samples.

MC exhibited a high *I*_D_/*I*_G_ ratio (0.65; Table 2). As previously discussed about fluorescence, this result suggests a higher content of sp^3^-hybridized carbon structures and/or structural defects, which explains the increased ratio compared to MAC-700-CO_2_ and MAC-700-N_2_, for instance. This interpretation is further supported by the relative intensities of the G^–^ and G^+^ bands: MC displays a more intense G^–^ band than G^+^ (Figure S5d), whereas MAC-700-CO_2_ (Figure S5f) and MAC-700-N_2_ (Figure S5i) show significantly stronger G^+^ bands.

**Table 2:** Ratio of the intensities of D_1_ e G^+^ bands obtained (*I*_D_/*I*_G_) from the deconvoluted spectra of MC, MAC-500-CO_2_, MAC-700-CO_2_, MAC-900-CO_2_, MAC-500-N_2_, MAC-700-N_2_, and MAC-900-N_2_

Samples	*I*_D_/*I*_G_
**MC**	0.65
**MAC-500-CO**_**2**_	0.29
**MAC-700-CO**_**2**_	0.37
**MAC-900-CO**_**2**_	1.46
**MAC-500-N**_**2**_	0.27
**MAC-700-N**_**2**_	0.42
**MAC-900-N**_**2**_	0.68

*I*_D_/*I*_G_ ratio also indicated that temperature influences the structure of the carbonaceous phase, as an increase in this ratio was observed when the temperature was raised from 500 to 900 °C under both atmospheres (Table 2). Furthermore, in the deconvoluted spectra of these samples (Figure S5e–j), the G^+^ band appeared more intense than the G^–^ band, suggesting that the thermal treatment under these conditions promoted surface functionalization (evidenced by the increased iron content, Table 1) without compromising the graphitic domains. The only exception was the sample treated at 900 °C under CO_2_ atmosphere (MAC-900-CO_2_). Its spectrum (Figure S5g) showed a marked reduction in the G^+^ and D_1_ bands and a sharp increase in the G^–^ band intensity. As shown in Table 1, this sample presented a lower carbon content due to degradation, which explains the high *I*_D_/*I*_G_ ratio (1.46). Therefore, when comparing the spectra of the samples treated at 900 °C in CO_2_ (MAC-900-CO_2_, Figure S5g) and N_2_ (MAC-900-N_2_, Figure S5j) atmospheres, it is evident that, as expected, thermal treatment in N_2_ prevents the degradation of the carbonaceous phase, preserving the graphitic domains.^55^

#### 2.1.2 Morphological and Textural Analysis

MC exhibited a specific surface area up to 15 times lower than that of the MACs (Table 3). In general, increasing the activation temperature (500 and 700 °C) led to higher specific surface areas. However, materials activated at 900 °C deviated from this trend. This behavior can be attributed to excessively high temperatures causing pore widening and collapse of micro- and mesopores into macropores, which contribute less to the overall surface area.^56^ Similar findings have been reported in other studies, which show a decreasing trend in surface area for activation temperatures above 800 °C.^28,56^

**Table 3:** Specific area (*S*_BET_), average pore diameter (*D*_p_), and total pore volume (*V*_t_) obtained from the N_2_ adsorption–desorption isotherms for MC and MACs

	Textural properties		
Samples	S_BET_ ^a^ (m^2^ g^–1^)	Pore diameter (*d*_p_)^b^ (nm)	Total pore volume (*V*_t_)^b^ (cm^3^ g^–1^)
**MC**	16	3.40	0.07
**MAC-500-CO**_**2**_	24	3.06	0.06
**MAC-700-CO**_**2**_	126	3.80	0.12
**MAC-900-CO**_**2**_	11	3.06	0.03
**MAC-500-N**_**2**_	66	3.80	0.09
**MAC-700-N**_**2**_	241	3.78	0.25
**MAC-900-N**_**2**_	227	3.78	0.27

a Total specific surface area was calculated using the BET method.

b Calculated using the BJH method.

For MAC-900-CO_2_, the specific surface area was even lower than that of the MC itself, suggesting that the oxidizing CO_2_ atmosphere intensified carbon phase degradation, leading to near-complete pore destruction, as indicated by the sharp decline in total pore volume (Table 3). Additionally, materials activated under N_2_ atmosphere exhibited higher surface areas than those activated in CO_2_, likely due to the inert nature of N_2_, which helps preserve micro- and mesopores by limiting carbon phase degradation, as already reported.^57–59^

N_2_ adsorption–desorption isotherms of MC are type III (Figure S6), according to the IUPAC classification, and are characterized by weaker adsorbent–adsorbate interactions than those between the adsorbate molecules, commonly associated with macroporous or nonporous materials.^60^ On the other hand, the activated materials exhibited type IV­(a) isotherms (Figure S6), typical of mesoporous materials, with capillary condensation accompanied by hysteresis, suggesting the presence of pores with a diameter greater than the critical width required for this phenomenon.^60^ The materials activated at 700 °C had the largest specific surface areas within their respective activation atmospheres (CO_2_ and N_2_), reaching 126 m^2^ g^–1^ and 241 m^2^ g^–1^, respectively. Therefore, they were selected for further analysis.

SEM images (Figure 3a) revealed particles with a morphological structure characteristic of lignocellulosic materials, similar to that observed in the study by Rattanachueskul et al.^61^ After HTC, MC showed agglomerates of particles (in the order of micrometers) with irregular morphologies (Figure 3b). Increasing the activation temperature to 700 °C in a CO_2_ and N_2_ atmosphere (Figure 3c,d, respectively) did not result in significant changes in particle morphology.

**Figure 3:**
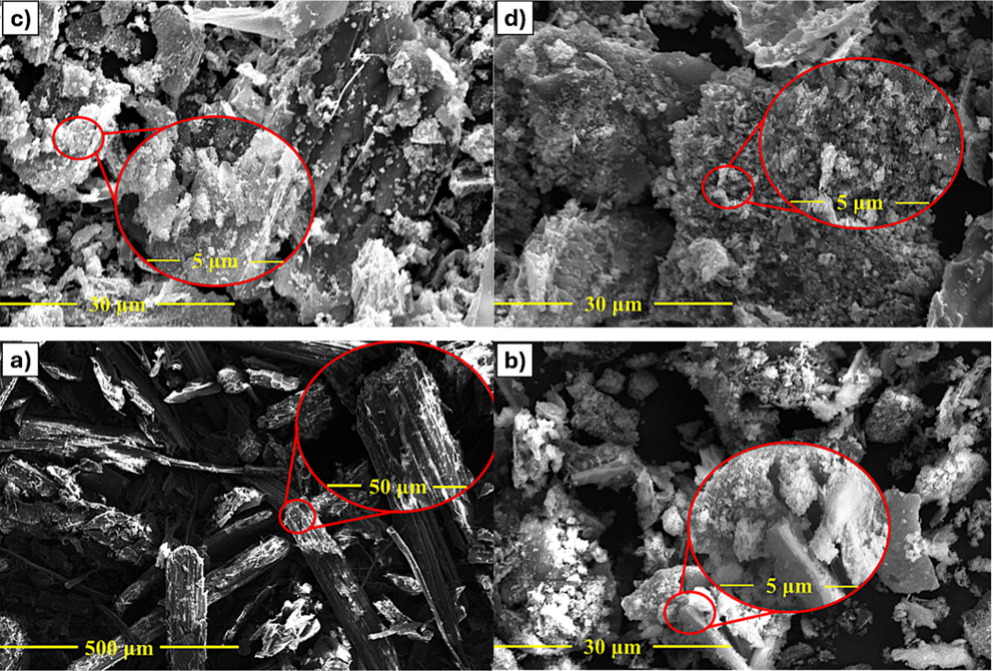
SEM images of (a) bagasse (500 and 50 μm), (b) MC (30 and 5 μm), (c) MAC-700-CO_2_ (30 and 5 μm) and (d) MAC-700-N_2_ (30 and 5 μm).

Elemental mapping analysis revealed a homogeneous distribution of carbon and oxygen in the precursor (sugar cane bagasse) (Figure S3a). After HTC (Figure S3b), iron was also uniformly incorporated. However, thermal activation at 700 °C under a CO_2_ atmosphere (Figure S3c) led to a reduction in the homogeneity of the element distribution, with agglomerates characteristic of the presence of new iron phases, as already observed in the XRD analysis (Figure 1). On the other hand, activation at the same temperature but under an N_2_ atmosphere (Figure S3d) maintained a highly homogeneous elemental distribution. These results suggest that the CO_2_ atmosphere favored greater interaction between the elements, which could lead to a redistribution of the elements. In contrast, the N_2_ atmosphere favored an inert environment, limiting surface reactivity and preserving elemental homogeneity.

#### 2.1.3 Surface Analysis: Determination of pH_zpc_

The pH_zpc_ of the MC was 6.68 (Table S1 and Figure 4). After activation at 700 °C in a CO_2_ and N_2_ atmosphere, there was a 1.1-fold increase in pH_zpc_ (7.22 and 7.24, respectively; Table S1 and Figure 4). This increase occurred because activation decreased oxygenated groups on the surface, a fact related to the high temperature used. The decrease in functional groups can also be seen in the FTIR analysis (Figure 2).

**Figure 4:**
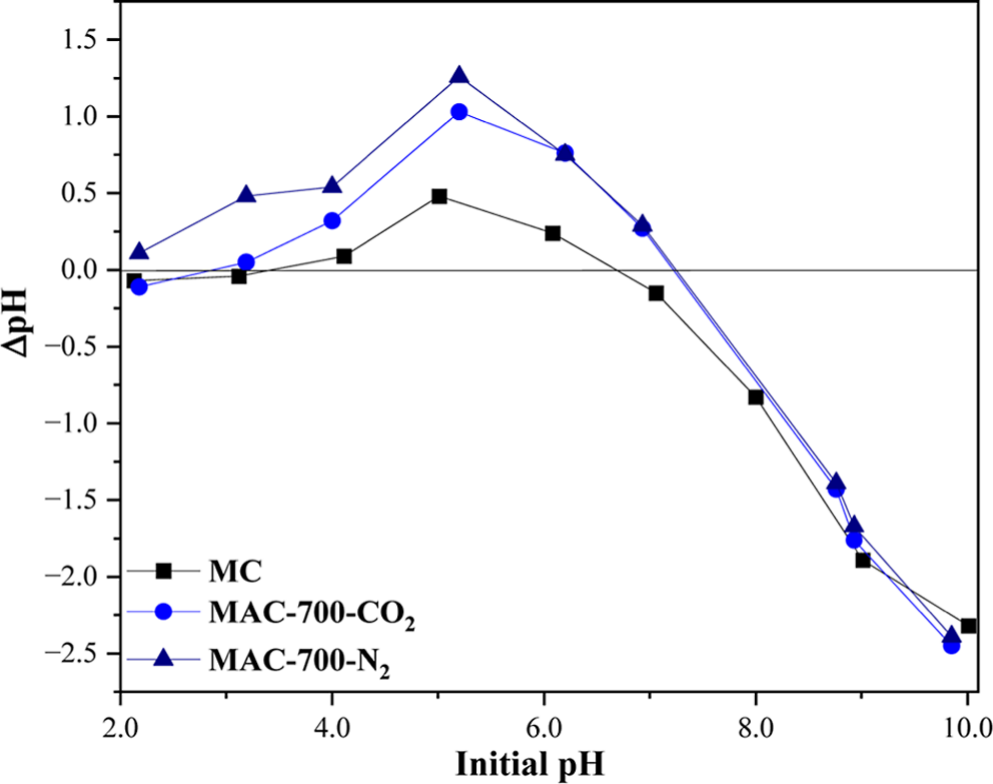
pH corresponding to the zero-charge point of MC, MAC-700-CO_2,_ and MAC-700-N_2_.

#### 2.1.4 Magnetic Analysis

MC, MAC-700-CO_2_, and MAC-700-N_2_ exhibited magnetic properties (Figure 5), with the activated materials being characterized by ferromagnetic behavior, as evidenced by the presence of hysteresis with magnetic remanence.^62–64^ This behavior is confirmed by the saturation magnetization (*M*_s_) values shown in Table S1, corresponding to 4.2, 29.1, and 27.4 emu g^–1^, respectively. These values are comparable to, and in some cases higher than, those observed in magnetic materials derived from agro-industrial wastes, such as pea and coconut shells, subjected to carbonization and activation processes (8–29 emu g^–1^).^14,39,65,66^ The data obtained indicate that activation in different atmospheres did not affect the magnetic properties of the materials but positively contributed to increasing their magnetization.

**Figure 5:**
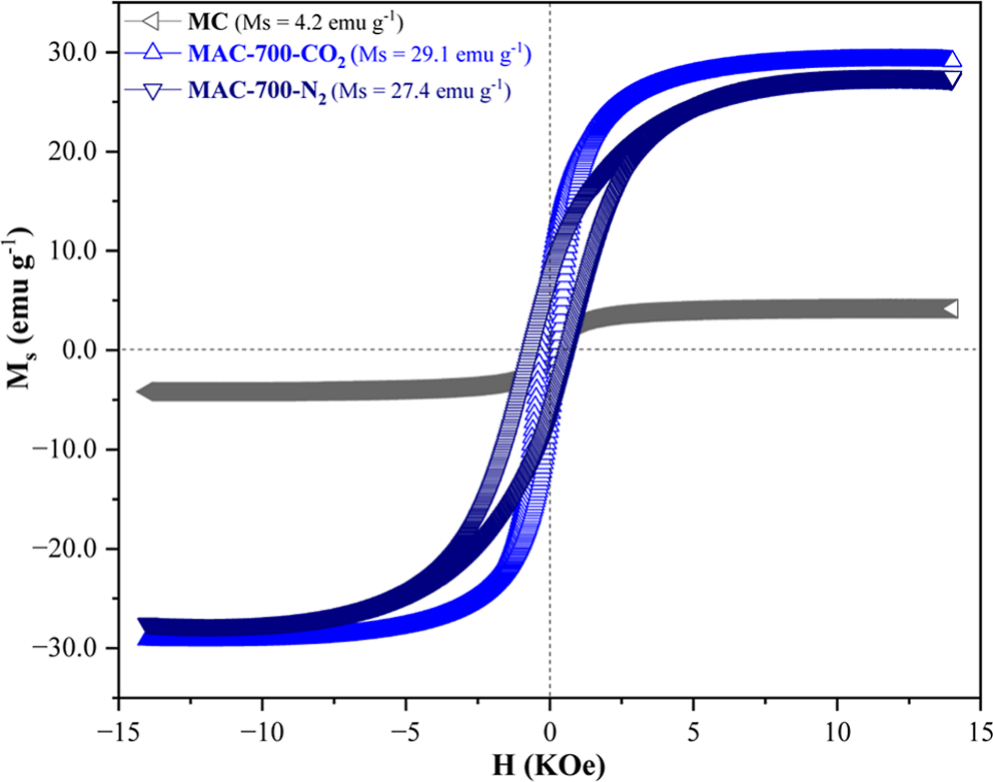
Magnetic hysteresis loops of MC, MAC-700-CO_2_, and MAC-700-N_2_.

XRD analysis showed the presence of ferromagnetic phases such as Fe_3_O_4_ and Fe^0^ (Figure 1). However, the *M*_s_ was probably reduced due to the possible encapsulation of the iron phases by carbon, which is diamagnetic.^67–69^ The narrow hysteresis indicates that these materials are soft and easy to magnetize.^16^ This highlights the potential application of these materials in processes requiring magnetic properties, such as magnetic separation.

### 2.2 MB and RB19 Adsorption

#### 2.2.1 Evaluation of the Adsorption of MB and RB19 by MC and MACs

MC could not adsorb any of the dyes evaluated (Figure 6a). Even after activation at 500 °C in a CO_2_ atmosphere, the behavior remained unchanged, with no significant adsorption (Figure 6a). On the other hand, activation at 500 °C in an N_2_ atmosphere resulted in the selective adsorption of the dye RB19, with an adsorbed amount of 6.5 mg g^–1^ (Figure 6a). When the activation temperature increased to 700 °C, in both CO_2_ and N_2_ atmospheres, there was a significant increase in the amount adsorbed, reaching up to 107 times more adsorption, with values of 17.9 and 74.0 mg g^–1^ for MB and 23.1 and 106.8 mg g^–1^ for RB19, respectively (Figure 6a). However, when the temperature increased to 900 °C in the CO_2_ and N_2_ atmospheres, there was a decrease in the amount adsorbed compared to the activation at 700 °C (Figure 6a).

**Figure 6:**
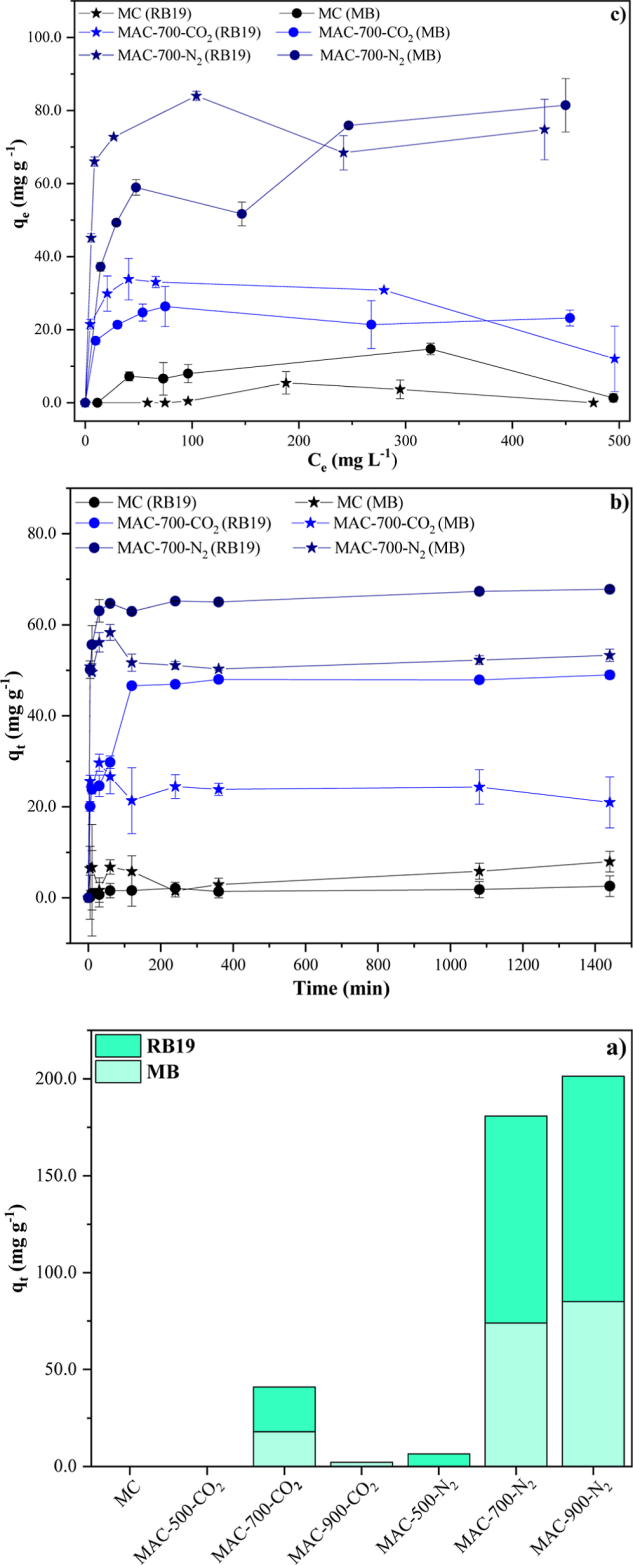
(a) Amounts of MB and RB19 adsorbed by MC, MAC-500-CO_2_, MAC-500-N_2_, MAC-700-CO_2_, MAC-700-N_2_, MAC-900-CO_2,_ and MAC-900-N_2_. Contact time of 1440 min, initial concentration of MB and RB19 of 250 mg L^–1^, and adsorbent dose of 1.0 g L^–1^; (b) amounts of MB and RB19 adsorbed by MC, MAC-700-CO_2,_ and MAC-700-N_2_ as a function of time (0–1440 min). Adsorbent dose of 1.0 g L^–1^ and initial concentration of MB and RB19 of 80.0 mg L^–1^; (c) amounts of MB and RB19 adsorbed by the MC, MAC-700-CO_2,_ and MAC-700-N_2_ as a function of the initial concentrations of MB and RB19 (0.0–500.0 mg L^–1^). Adsorbent dose of 1.0 g L^–1^ and contact time of 240 min. The experiments were carried out at a temperature of ∼22 °C.

Increasing the activation temperature, especially in different atmospheres, favors the development of a more accessible porous structure and generates active sites, increasing the amount adsorbed.^17,23,57^ However, activation at higher temperatures, such as 900 °C, can lead to pore destruction, a result confirmed by the textural properties (Table 3). In this scenario, the increase in temperature causes the pores to expand, destroying the walls between adjacent pores and consequently widening the pores, which decreases the amount adsorbed, as observed in other studies.^57,70,71^

Adsorption increased up to 38 times for materials activated in an N_2_ atmosphere compared to those activated in a CO_2_ atmosphere (Figure 6a). To elucidate the effects of the different activation atmospheres on the adsorbent properties, kinetic and isothermal adsorption studies were performed for the dyes MB and RB19 using only MC and the activated materials MAC-700-CO_2_ and MAC-700-N_2_, which exhibited the highest adsorption capacities on each set of adsorbents. The selection of these materials was based on their effectiveness in adsorbing both dyes, as well as the objective of evaluating the effect of different activation atmospheres.

#### 2.2.2 MB and RB19 Adsorption Kinetics

Analysis of the adsorption kinetics showed that MC did not interact effectively with MB and RB19, as there was no variation in the amount of these dyes adsorbed over the contact time (Figure 6b). For MAC-700-CO_2_ and MAC-700-N_2_, the adsorption of MB and RB19 occurred rapidly in the first 100 min, reaching a plateau at 240 min, after which there were no relevant changes in the amount adsorbed (Figure 6b), which was considered the equilibrium time used in isothermal studies (Figure 6c). Initially, the available pores provided a high number of active sites for adsorbate–adsorbent interactions, facilitating the adsorption of the dye molecules. With time, the pores gradually saturate, resulting in a decrease in the number of available sites and making it difficult to adsorb additional molecules. This behavior can be attributed to the repulsive effect between the already adsorbed MB or RB19 molecules and those present in the liquid phase, which triggers a process of continuous adsorption and desorption until the system is in equilibrium. Similar adsorption patterns have been reported in studies using different adsorbents, suggesting that the saturation of the active sites and the interaction forces between the adsorbed molecules are the determining factors for equilibrium.^72–74^

To investigate the adsorption kinetics, the pseudo-first-order (PFO) and pseudo-second-order (PSO) models were applied, along with the intraparticle diffusion (ID) and diffusion-chemisorption (DC) models, to assess their fit to the experimental data (eqs S1–S4). The kinetic parameters obtained (Table S3), in conjunction with the error function adj. *R* ^2^, indicated that the adsorption of MB onto the three materials was governed by physical, chemical, and diffusion processes. This finding is supported by the closely related parameter values, which suggest a mixed adsorption mechanism.^75,76^ On the other hand, the kinetic parameters and error functions for RB19 adsorption indicated a predominance of chemical and diffusion processes, suggesting a more homogeneous adsorption mechanism.^75,76^ However, due to the similarity of the parameter values, it is not feasible to distinguish the adsorption mechanism for the three materials and the two dyes, as it is likely mixed.

#### 2.2.3 MB and RB19 Adsorption Isotherms

A thorough examination of the MC adsorption isotherm indicated that, despite the rise in MB and RB19 concentrations, there was no concomitant increase in the material’s adsorption capacity (Figure 6c). However, the activation of the material at 700 °C in a CO_2_ atmosphere led to a 1.8-fold and 6.2-fold increase, respectively, in the adsorption capacity of MB and RB19 (Figure 6c). Upon activation in an N_2_ atmosphere, the material demonstrated a substantial enhancement in adsorption capacity, exhibiting an approximate increase of 5.5 and 15.5 times for MB and RB19, respectively (Figure 6c).

The classical Langmuir and Freundlich isotherm models, along with the hybrid Sips model (eqs S5–S7), were applied to identify the best fit to the experimental data. The isotherm parameters and error function (adj. *R* ^2^, Table S4) indicated that the Sips model provided the best fit for MB and RB19 adsorption across all materials, suggesting a mixed adsorption mechanism. This is consistent with the kinetic results, which showed very similar parameter values. The Sips model is typical for surfaces with heterogeneous adsorption energies and effectively describes adsorption behavior at both low and high adsorbate concentrations.^75,77^

The final adsorption pHs of MB and RB19 were lower than pH_zpc_ (Table S1), indicating that electrostatic favoring would only occur for the adsorption of RB19, which is anionic. However, as there were similar adsorption capacities for both dyes, adsorption did not occur primarily through electrostatic adsorbent–adsorbate mechanisms. Overall, the increase in specific surface area and pore volume played a key role in adsorption performance, as evidenced by the superior results for MAC-700-N_2_ compared to MAC-700-CO_2_ (Table 3). N_2_ adsorption–desorption isotherms (Figure S6) revealed that the materials are predominantly mesoporous, which facilitates the diffusion of medium-to-large-sized molecules such as the dyes evaluated.

MB and RB19 have molecular weights of 319.8 and 626.5 g mol^–1^, respectively.^78,79^ Although MB is smaller, its diffusion in mesoporous materials may be limited due to weaker interactions with pore surfaces, potentially hindering retention. In contrast, RB19’s size and structure allow better penetration and stronger interactions with the pore surfaces, resulting in higher adsorption. Nevertheless, the adsorption capacities of MB and RB19 were similar (26.3 and 30.8 mg g^–1^ for MAC-700-CO_2_; 81.4 and 74.8 mg g^–1^ for MAC-700-N_2_, respectively), demonstrating the multifunctionality of the materials in adsorbing both cationic and anionic dyes. The activation atmosphere played a critical role in pore development, with N_2_ proving more effective. Activation under N_2_ prevents excessive oxidation of the carbon matrix, favoring pore formation and preservation, as reflected in the higher specific surface area and pore volume (Table 3).

A comparison of the MB and RB19 adsorption capacities of MAC-700-CO_2_ and MAC-700-N_2_ with literature-reported materials is shown in Table 4. The results demonstrate that both materials exhibit adsorption capacities comparable to previously reported sorbents, highlighting thermal activation under CO_2_ and particularly N_2_ as promising routes for developing sustainable materials for cationic and anionic dye removal. The adsorption observed in the study using residues from the production of 5-Sodium sulfodimethyl isophthalate (Table 4) showed an exceptional adsorption capacity.^80^ However, this material was produced by a different route and precursor, as well as under adsorption conditions different from those used in this work. Thermal activation also offers environmental advantages, such as reduced waste generation, positioning it as a viable alternative to chemical or thermochemical activation methods.

**Table 4:** Comparative analysis conducted between the adsorption capacity obtained in other studies and the adsorption apacity of the dyes MB and RB19 in this study, obtained by MAC-700-N_2_ and MAC-700-CO_2_

Synthesis precursor	Activation conditions	Dye	Removal capacity	refs
Tire-derived activated carbon magnetized with Fe_3_O_4_ particles	Chemical activation with H_3_PO_4_, 500 °C for 100 min	RB19	119.0 mg g^–1^	^81^
Industrial laundry sewage sludge mesoporous activated carbon	Thermal activation with CO_2_, 750 °C for 60 min	RB19	33.5 mg g^–1^	^82^
Sugar cane bagasse and ferric nitrate magnetic activated carbon	Thermal activation with CO_2_, 700 °C for 60 min	RB19	30.8 mg g^–1^	this stud*y*
	Activation at N_2_, 700 °C for 60 min		74.8 mg g^–1^	
Sewage sludge activated carbon	CO_2_ thermal activation, 800 °C for 30 min	MB	30.2 mg g^–1^	^83^
Activated carbon derived from bamboo biomass	Chemical activation by KOH, 900 °C for 120 min	MB	83.3 mg g^–1^	^84^
Waste liquid produced in the production process of 5-sodium sulfodimethyl isophthalate	Drying to constant weight and calcination in a muffle furnace for 120 min	MB	2816.4 mg g^–1^	^80^
Sugar cane bagasse and ferric nitrate magnetic activated carbon	Thermal activation with CO_2_, 700 °C for 60 min	MB	23.2 mg g^–1^	this study
	Activation at N_2_, 700 °C for 60 min		81.4 mg g^–1^	

In summary, the results demonstrate that thermal activation of magnetic carbons derived from hydrothermal carbonization of sugar cane bagasse under CO_2_ and N_2_ atmospheres is an effective strategy for optimizing structural and magnetic properties without the use of additional chemical agents. Activation at 700 °C proved particularly effective, achieving a balance between high surface area and magnetization, key attributes for environmental applications. Although dye adsorption was employed as a proof of concept, the primary aim of this study was to develop multifunctional materials with potential for various applications, such as catalysis or magnetic separation. Thus, this work offers a sustainable route for producing advanced magnetic carbon materials from agricultural waste, aligning functional performance with the principles of green chemistry.

## 3 Conclusion

MC was successfully synthesized via HTC of sugar cane bagasse with ferric nitrate at 270 °C, followed by thermal activation under CO_2_ and N_2_ atmospheres at 500, 700, and 900 °C. Activation at 700 °C was the most effective condition, with CO_2_ favoring the formation of magnetite and N_2_ yielding iron carbide and zero-valent iron. This thermal treatment significantly improved the materials’ textural and magnetic properties, with specific surface areas of 126 m^2^ g^–1^ (CO_2_) and 241 m_2_ g^–1^ (N_2_), magnetization values of 29.1 emu g^–1^ (CO_2_) and 27.4 emu g^–1^ (N_2_), and low *I*_D_/*I*_G_ ratios (0.37 for CO_2_ and 0.42 for N_2_), indicating a predominance of graphitic domains. These features enhanced the adsorption of MB (81.4 mg g^–1^) and RB19 (74.8 mg g^–1^), demonstrating the materials’ capacity to remove both cationic and anionic dyes. Kinetic analyses revealed a mixed adsorption mechanism for MB, involving physical, chemical, and intraparticle diffusion processes, while RB19 adsorption was primarily governed by chemical interaction and diffusion. The similarity in kinetic parameters suggested no clear mechanistic distinction between the materials. The Sips model best fitted the equilibrium data for both dyes, supporting the coexistence of heterogeneous adsorption sites. Overall, thermal activation at 700 °C, particularly under N_2_, proved to be the most favorable strategy for enhancing MC performance. These results meet the study objective of developing high surface area and magnetically separable carbon materials through a sustainable process, reinforcing their potential multifunctional application for cationic and anionic dye removal.

## 4 Experimental Section

### 4.1 Chemicals and Materials

Sugar cane bagasse was collected from the sugar cane industry in the São José do Rio Preto region, São Paulo, Brazil. The material was washed with distilled water, dried in an oven at 100 °C for 12 h (Tecnik’s, NL80/10CEL), ground using a knife mill (Marconi, MA 340), and sieved through a 0.25 mm mesh (Bertel). The resulting material was then stored at room temperature. The reagents ferric nitrate nonahydrate (Fe­(NO_3_)_3_·9H_2_O, 99.0%), hydrochloric acid (HCl, 37.0%), sodium hydroxide (NaOH, 99.0%), and sodium chloride (NaCl, 99.0%) were purchased from Synth. The dyes MB (C_16_H_18_ClN_3_S) and RB19 (C_22_H_16_N_2_Na_2_O_11_S_3_) were obtained from Sigma-Aldrich. All solutions were prepared using Milli-Q ultrapure water (18.2 MΩ cm^–1^). Carbon dioxide (CO_2_, 99.8%) and nitrogen (N_2_, 99.9%) gases were supplied by White Martins.

### 4.2 MC Production and Thermal Activation under N_2_ or CO_2_ Atmosphere

MC was produced by HTC of sugar cane bagasse in the presence of ferric nitrate nonahydrate (Fe­(NO_3_)_3_·9H_2_O) at 270 °C, following the procedure described in a previous study.^10^ After HTC, the MC was washed with deionized water under vacuum filtration until reaching a pH of ∼5.0. The resulting solid was then dried in an oven at 65 °C for 24 h, ground, sieved (0.25 mm, Bertel), and stored at room temperature.

Thermal activation was performed by adapting procedures reported in previous studies,^30,85^ adding 0.50 g of MC to an alumina boat, which was inserted into a tubular furnace (EDG, FT-HI/20/Bipartite) and exposed to 400 mL min^–1^ of CO_2_ or N_2_, heated at 10 °C min^–1^ to 500, 700, or 900 °C, remaining at this temperature for 1 h. The furnace was then cooled under the flow of activation gas, and the samples were removed at room temperature, then macerated, sieved (0.15 mm, Bertel), and stored. The activated samples were named following the “*MAC-T*_*a*_ *-g*” pattern, where “*T*_*a*_” denotes the activation temperature and “*g*” represents the gas used in the activation.

### 4.3 Characterizations

The MC used in this study for subsequent activation was developed based on a previous study,^10^ in which its composition, structure, morphology, texture, and surface were characterized. Since the material had to be produced from sugar cane bagasse collected at a different time, and given the high heterogeneity of this biomass, the analyses were repeated in the present study. The MACs presented in this work were produced for the first time under these conditions.

#### 4.3.1 Compositional and Structural Analysis

CHN Elemental analysis of MC and MACs was performed using a CHN Elemental Analyzer (Fisons Instruments, EA1108) to determine the carbon (C), hydrogen (H), and nitrogen (N) contents. Ash content in these materials was determined by thermogravimetric curves (TG) over a temperature range of 30.0 to 900.0 °C, with a heating rate of 10.0 °C min^–1^ under a synthetic air flow of 50.0 mL min^–1^. Surface functional groups of MC and MACs were analyzed by Fourier-transform infrared spectroscopy (FTIR) using an attenuated total reflectance (ATR) accessory (PerkinElmer, Spectrum Two). Spectra were acquired in the 4000–400 cm^–1^ range at a resolution of 2 cm^–1^ with 128 scans in absorbance mode (Bruker, Vertex 70), and then converted to transmittance mode. Raman spectra were obtained using a portable spectrometer (Anton Paar, DeltaNu Advantage532) with a spectral resolution of 8 cm^–1^, laser power of 23.3 mW, and excitation wavelength of λ = 532 nm. Measurements were performed using 10 scans with an integration time of 40 s. Spectra were deconvoluted using the Voigt function to fit the curves,^52^ considering the D_1_, D_2_, G-, and G+ components, the latter two also referred to as G_1_ and G_2_.^86,87^ Inorganic phases in the materials were identified by X-ray diffraction (XRD) (Rigaku, Miniflex 300), using Cu–Kα radiation (λ = 1.54 Å) generated at 40 mA and 40 kV. Scans were conducted at a rate of 0.02° min^–1^ over the 2θ range of 10–85°. Phase identification was performed using crystallographic data from the international centre for diffraction data (ICDD).

#### 4.3.2 Morphological and Textural Analysis

Morphology of the samples was evaluated using a scanning electron microscope (FEI, Helios Nanolab 6000) equipped with secondary electron (SE) detectors and an energy-dispersive X-ray spectroscopy (EDS) microanalysis system. For textural analysis, approximately 0.20 g of each sample was pretreated in a degasser at 150 °C for 3 h under vacuum. Nitrogen adsorption–desorption analyses (Quantachrome, Nova 1200) were subsequently performed at −196.2 °C over a relative pressure range of 0.050 to 0.995 (*p*/*p*_0_) to determine the specific surface area, pore diameter, and pore volume of the MC and MACs. The specific surface area was calculated using the Brunauer–Emmett–Teller (BET) method, while pore diameter and volume were determined using the Barrett–Joyner–Halenda (BJH) model.

#### 4.3.3 Determination of pH Corresponding to the Zero-Charge Point

Experiments were carried out to determine the pH corresponding to the zero-charge point (pH_zpc_) using NaCl 0.01 mol L^–1^ as the electrolyte at 9 pH points ranging from 2.0 to 10.0.^10,88^ The pH adjustment was performed using 0.1 mol L^–1^ HCl or NaOH, and the adjusted solutions were added to the materials in Erlenmeyer flasks at a dosage of 0.50 g L^–1^.

#### 4.3.4 Analysis of Magnetic Properties

Magnetic hysteresis curves were measured using a vibrating sample magnetometer (VSM; Princeton Measurement Corp, 2900/3900-02 AGM/VSM) at room temperature (approximately 26.8 °C). The procedure involved applying an increasing magnetic field from 0 to 1 T, with continuous magnetization measurements recorded at 2 mT intervals. Subsequently, the magnetic field was systematically decreased to −1 T and then increased again to 1 T, completing a full hysteresis loop.

### 4.4 MB and RB19 Adsorption

MB and RB19 solutions were prepared from a stock solution of 1250 mg L^–1^ for all adsorption experiments. After the stirring period, the samples were filtered through PVDF membranes (0.22 μm, Millipore). The dyes were analyzed using a UV–vis molecular absorption spectrophotometer (Shimadzu, UV-2600) at wavelengths of 664 nm (MB) and 595 nm (RB19).

#### 4.4.1 Evaluation of the Adsorption of MB and RB19 by MC and MACs

The adsorbents MC, MAC-500-CO_2_, MAC-500-N_2_, MAC-700-CO_2_, MAC-700-N_2_, MAC-900-CO_2_, and MAC-900-N_2_ were evaluated for adsorption in aqueous solutions with a concentration of 250 mg L^–1^ of MB and RB19 dosage of 1.0 g L^–1^, stirred at 180 rpm for 1440 min. The adsorbed amounts (*q*_t_) of MB and RB19 were calculated using eq 6.

6 $$q_{t}=\frac{\left(c_{0}-c_{t}\right)\times V}{m}$$

where “*V*” is the volume of MB and RB19 solution used (L), “*m*” is the mass of the adsorbent (g), and “*C*_0_” and “*C*_*t*_” are the initial and at time “*t*” concentrations, respectively.

#### 4.4.2 MB and RB19 Adsorption Kinetics

Equilibrium time of the MC, MAC-700-CO_2_, and MAC-700-N_2_ (selected in Section 4.4.1) was evaluated using a dosage of 1.0 g L^–1^. The carbons were mixed with MB and RB19 dyes at a concentration of 80.0 mg L^–1^ in Erlenmeyer flasks, subjected to ultrasonic treatment, and maintained under continuous stirring at 180 rpm for 0, 5, 10, 30, 60, 120, 240, 360, 1080, and 1440 min. The amounts adsorbed (*q*_t_) of MB and RB19 were calculated using eq 6. The results were fitted to the nonlinear kinetic models of Pseudo-First Order (PFO–Equation S1), Pseudo-Second Order (PSO–Equation S2), Intraparticle Diffusion (ID–Equation S3), and Diffusion-Chemissorption (DC–Equation S4).^76,89^

#### 4.4.3 MB and RB19 Adsorption Isotherms

Adsorption capacities of MC, MAC-700-CO_2_, and MAC-700-N_2_ were determined using a dosage of 1.0 g L^–1^, while varying the concentrations of MB and RB19 at 0.0, 25.0, 50.0, 75.0, 100.0, 200.0, 300.0, and 500.0 mg L^–1^. The samples were stirred at 180 rpm for 240 min (Section 4.4.2). The adsorption capacity (*q*_e_) was calculated using eq 7.

7 $$q_{e}=\frac{\left(c_{0}-c_{e}\right)\times V}{m}$$

where “*V*” is the volume of MB and RB19 solution used (L), “*m*” is the mass of the adsorbent (g), and “*C*_0_” and “*C*_*e*_” represent the initial and equilibrium concentrations, respectively. The adsorption isotherm of each material was evaluated using the nonlinear Langmuir (Equation S5), Freundlich (Equation S6), and Sips (Equation S7) models.^77,90^

## Supplementary Material



## Data Availability

The data underlying this study are openly available in UNESP Institutional Repository at https://hdl.handle.net/11449/255825.

## Notes

Mathematical equations of kinetic and isotherm models; pH_zpc_ and final pH values for MB and RB19 adsorption; magnetic properties (H_c_, M_r_, M_s_) from VSM analysis; kinetic parameters and error functions for PFO, PSO, ID, and DC models; isotherm fit parameters for Langmuir, Freundlich, and Sips models; thermogravimetric profiles of raw and MACs; XRD patterns of sugar cane bagasse and MC; SEM images and elemental mapping of Fe, C, and O in raw and MACs; FTIR and Raman spectra (including deconvoluted spectra) of MC and MACs; N_2_ adsorption–desorption isotherms at – 196 °C; nonlinear kinetic and isotherm model fitting plots for MB and RB19 (PDF)
