# Supplementary material for: Effect of the Thermal Activation on the Adsorption Capacity of Cationic and Anionic Dyes in Magnetic Carbon
Source: ACS Omega. 2025 Aug 5;10(32):36631–43. doi: 10.1021/acsomega.5c05783 (PMC12368825; doi:10.1021/acsomega.5c05783)
Supplement: Supplementary file 1 [file ao5c05783_si_001.pdf]

SUPPLEMENTARY DATA

Effect of the Thermal Activation on the Adsorption Capacity of Cationic and Anionic Dyes in Magnetic Carbon

Gabriela A. Nogueira[a], Jaqueline R. Lopes[a], Eduardo C. Vilas Boas[b], Thiago N. M. Cervantes[c], Altair B. Moreira[a], Odair P. Ferreira[b], Márcia C. Bisinoti[a,*]

[a] *São Paulo State University (UNESP), Institute of Biosciences, Letters and Exact Sciences (IBILCE), Department of Chemistry and Environmental Sciences (DQCA), Laboratory of Environmental Sciences Studies (LECA), 2265 Cristóvão Colombo St., Jardim Nazareth, São José do Rio Preto – SP, ZIP code 15054-000, Brazil.* gabriela.aparecida@unesp.br (G.A.N.); jr.lopes@unesp.br (J.R.L.); altair.moreira@unesp.br (A.B.M.).

[b] *State University of Londrina (UEL), Department of Chemistry, Advanced Functional Materials Laboratory (LaMFA), Celso Garcia Cid Highway, PR 445, km 380, Londrina – PR, ZIP code 86055-900, Brazil.* thiagocervantes@uel.br *(T.N.M.C.).*

[c] *State University of Londrina (UEL), Department of Chemistry, Laboratory of Environmental Electrochemistry (LabEA), Celso Garcia Cid Highway, PR 445, km 380, Londrina – PR, ZIP code 86055-900, Brazil.* eduardo.custodio@uel.br (E.C.V.B.); opferreira@uel.br (O.P.F.).

**Corresponding author:* marcia.bisinoti@unesp.br.

I) Equations

The kinetic data of the MB and RB19 adsorption experiment were evaluated, according to pseudo-first order (PFO; Equation S1), pseudo-second order (PSO; Equation S2), intraparticle diffusion (ID; Equation S3), and diffusion-chemisorption (DC; Equation S4) non-linear models.

$$q_t = q_e[1 - e^{(-k_1 t)}] \tag{S1}$$

$$q_t = \frac{k_2(q_e)^2 t}{1 + k_2 q_e t} \tag{S2}$$

$$q_t = k_{ID}\sqrt{t} + C \tag{S3}$$

$$q_t = \frac{q_e k_{DQ}\sqrt{t}}{k_{DC}\sqrt{t} + q_e} \tag{S4}$$

where "q_e" (mg g^{-1}) and "q_t" (mg g^{-1}) represent the amounts of MB and RB19 adsorbed at equilibrium and at time "t" (min), respectively; "k_1" (min^{-1}) and "k_2" (g mg^{-1} min^{-1}) are the rate constants of PFO and PSO models, respectively; "k_{ID}" (mg g^{-1} min^{0,5}) is the intraparticle diffusion rate constant; "C" (mg g^{-1}) is a constant related to the thickness of the adsorption boundary layer; "k_{DC}" (mg g^{-1} min$^{-0,5}$) is a diffusion-chemisorption constant.

The isotherm data of the MB and RB19 adsorption experiment were evaluated, according to Langmuir (Equation S5), Freundlich (Equation S6), and Sips (Equation S7) non-linear models.

$$q_e = K_F \times C_e{}^{1/n} \tag{S5}$$

$$q_e = \frac{q_m \times K_L \times C_e}{1 + K_L \times C_e} \tag{S6}$$

$$q_e = \frac{q_{ms} \times K_s \times C_e{}^{ns}}{1 + K_s \times C_e{}^{ns}} \tag{S7}$$

where "q_e" is the amount of MB and RB19 adsorbed at equilibrium (mg g^{-1}), "k_F" (L$^{1/n}$ mg$^{(1-1/n)*-1}$) and "n" are the Freundlich model constants, "C_e" is the equilibrium concentration (mg L^{-1}), "q_m" is the estimated adsorption capacity (mg g^{-1}), "K_L" (L mg^{-1}) is the Langmuir constant, "q_{ms}" is the maximum adsorption capacity (mg g^{-1}), and "K_s" (L^{ns} mg^{-ns}) and "n_s" are the Sips model constants.

Assuming that the inorganic fraction remaining in the samples after TGA analysis consisted entirely of Fe_2O_3 (stable phase at the end of the process), the mass of Fe was estimated indirectly. The percentage of Fe was calculated from Equations S8 and S9.

$$m_{Fe} = \frac{m_f \times 2 \times MM_{Fe}}{MM_{Fe_2O_3}} \tag{S8}$$

$$Fe\ (\%) = \frac{m_{Fe} \times 100}{m_i} \tag{S9}$$

where "m_{Fe}" is the mass of Fe, "m_f" the final mass after TGA, and "m_i" the initial mass. The molar mass of Fe_2O_3 (159.7 g mol^{-1}) and that of Fe (55.8 g mol^{-1}).

II) Tables

Table S1. pH_{zpc} and final pH of MB and RB19 adsorption.

Samples	pH_{zpc}	Final pH (MB)	Final pH (RB19)
**MC**	6.68	5.38 ± 0.43	5.70 ± 0.11
**MAC-700-CO_2**	7.22	5.28 ± 0.15	6.71 ± 0.43
**MAC-700-N_2**	7.24	5.59 ± 0.23	6.74 ± 0.03

Table S2. Values of coercivity (Hc), remaining magnetization (Mr), and saturation magnetization (Ms) obtained in the VSM analysis of the MC and MAC-700-CO_2 and MAC-700-N_2.

Samples	Hc (kOe)	Mr (emu g^{-1})	Ms (emu g^{-1})
**MC**	0.09	0.7	4.2
**MAC-700-CO_2**	0.21	7.9	29.1
**MAC-700-N_2**	0.46	6.8	27.4

Table S3. Kinetic parameters extracted from the application of the PFO, PSO, ID, and DC models to the experimental MB and RB19 adsorption data for the MC, MAC-700-CO_2, and MAC-700-N_2, as well as the error functions.

Models	MC (MB)	MAC-700-CO_2 (MB)	MAC-700-N_2 (MB)	MC (RB19)	MAC-700-CO_2 (RB19)	MAC-700-N_2 (RB19)
**PFO**						
q_e (mg g^{-1})	5.1	24.5	52.6	1.9	37.4	62.4
K_1 (min^{-1})	$5.4*10^1$	$7.1*10^6$	$1.1*10^1$	$2.3*10^{-2}$	$0.8*10^1$	$1.2*10^1$
R^2	*0.335*	*0.907*	*0.974*	*0.798*	*0.505*	*0.928*
Adj R^2	*0.252*	*0.896*	*0.971*	*0.773*	*0.443*	*0.919*
**PSO**						
q_e (mg g^{-1})	5.1	24.5	53.1	2.1	48.1	66.1
K_2 (g mg^{-1} min^{-1})	$1.5*10^{17}$	$2.5*10^9$	$7.4*10^{-2}$	$1.6*10^{-2}$	$1.0*10^{-3}$	$9.0*10^{-3}$
R^2	*0.335*	*0.907*	*0.976*	*0.823*	*0.904*	*0.997*
Adj R^2	*0.252*	*0.896*	*0.973*	*0.800*	*0.892*	*0.996*
**ID**						
K_{ID} (mg g^{-1})	$7.7*10^{-2}$	$1.2*10^{-1}$	$4.6*10^{-1}$	$5.2*10^{-2}$	$9.9*10^{-1}$	$8.4*10^{-1}$
C (mg g^{-1})	3.5	20.5	41.2	0.6	20.2	44.9
R^2	*0.131*	*0.037*	*0.124*	*0.661*	*0.611*	*0.283*
Adj R^2	*0.022*	*-0.083*	*0.015*	*0.619*	*0.562*	*0.193*
**DC**						
K_{DQ} (mg g^{-1} $min^{-0.5}$)	$7.8*10^{43}$	$3.6*10^{15}$	$7.0*10^2$	$2.9*10^{-1}$	$1.2*10^1$	$9.2*10^1$
q_e (mg g^{-1})	5.1	24.5	53.2	2.8	57.6	68.9
R^2	*0.335*	*0.907*	*0.975*	*0.824*	*0.944*	*0.996*
Adj R^2	*0.252*	*0.896*	*0.972*	*0.802*	*0.937*	*0.995*

Table S4. Fit parameters of the Langmuir, Freundlich, and Sips isotherm models for the MB and RB19 adsorption isotherms for the MC and MAC-700-CO_2 and MAC-700-N_2 samples.

**Models**	**MC (MB)**	**MAC-700-CO_2 (MB)**	**MAC-700-N_2 (MB)**	**MC (RB19)**	**MAC-700-CO_2 (RB19)**	**MAC-700-N_2 (RB19)**
**Langmuir**						
q_m (mg g^{-1})	8.9	24.4	76.0	3.1	27.9	77.7
K_L (L mg^{-1})	$4.1*10^{-2}$	$2.7*10^{-1}$	$6.1*10^{-2}$	$8.0*10^{-3}$	$0.1*10^{1}$	$3.6*10^{-1}$
R^2	*0.398*	*0.954*	*0.915*	*0.200*	*0.646*	*0.949*
Adj R^2	*0.277*	*0.945*	*0.898*	*0.040*	*0.575*	*0.939*
**Freundlich**						
n_F	$0.6*10^{1}$	$2.1*10^{1}$	$0.5*10^{1}$	$0.3*10^{1}$	$1.5*10^{21}$	$1.4*10^{1}$
K_F ($L^{1/n}$ $mg^{1-1//n*-1}$)	2.8	18.2	24.1	0.3	26.9	52.5
R^2	*0.289*	*0.914*	*0.922*	*0.142*	*0.631*	*0.898*
Adj R^2	*0.147*	*0.897*	*0.910*	*-0.030*	*0.557*	*0.878*
**Sips**						
q_{ms} (mg g^{-1})	7.7	23.7	80.0	3.0	27.9	76.0
K_s (L^{ns} mg^{-ns})	$4.4*10^{-8}$	$2.5*10^{-2}$	$1.2*10^{-1}$	$3.6*10^{-17}$	$6.1*10^{-6}$	$5.0*10^{-3}$
n_s	5.2	2.0	0.7	8.0	8.6	3.3
R^2	*0.472*	*0.964*	*0.944*	*0.481*	*0.666*	*0.973*
Adj R^2	*0.208*	*0.947*	*0.936*	*0.221*	*0.499*	*0.960*

III) Figures

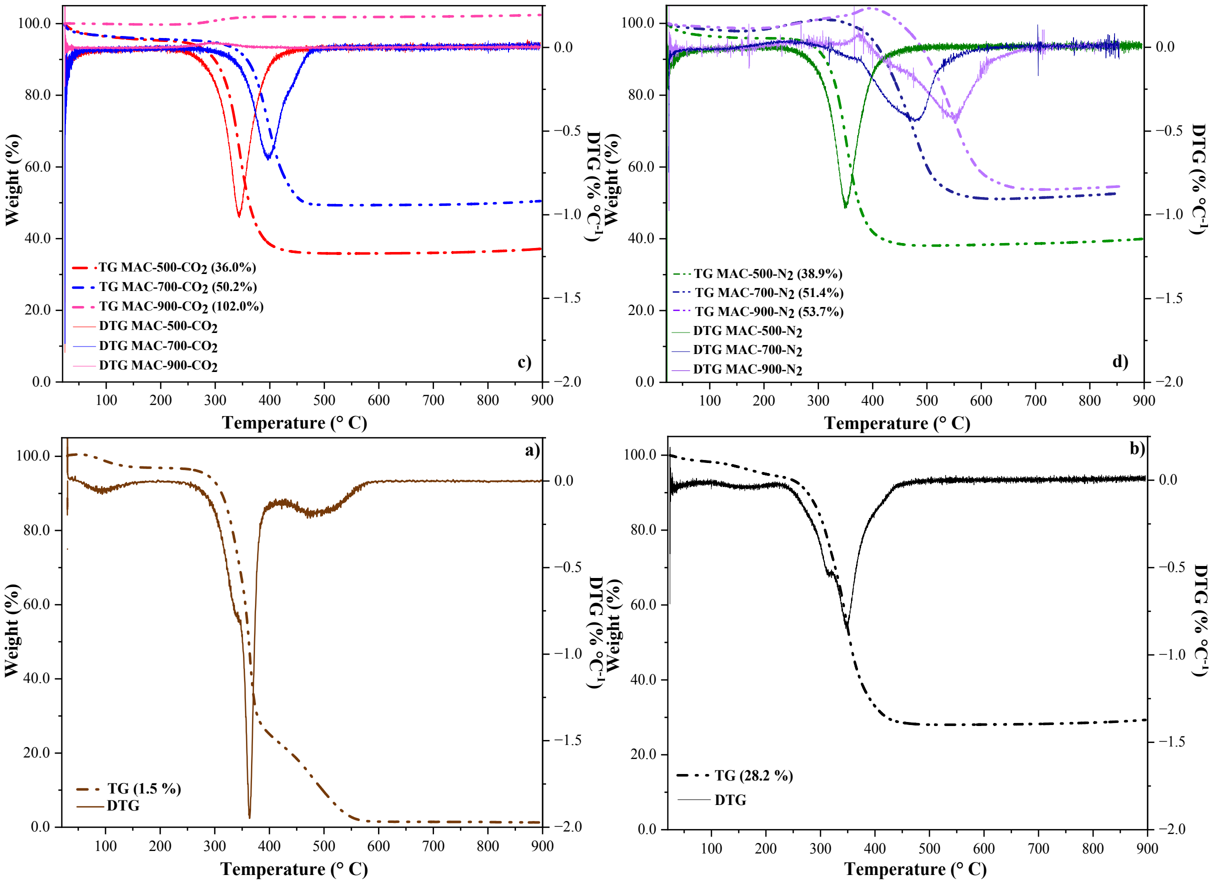

Figure S1. Thermogravimetric profiles (TG) corresponding to samples of a) sugarcane bagasse; b) MC; c) MAC-500-CO_2, MAC-700-CO_2, MAC-900-CO_2; d) MAC-500-N_2, MAC-700-N_2, and MAC-900-N_2.

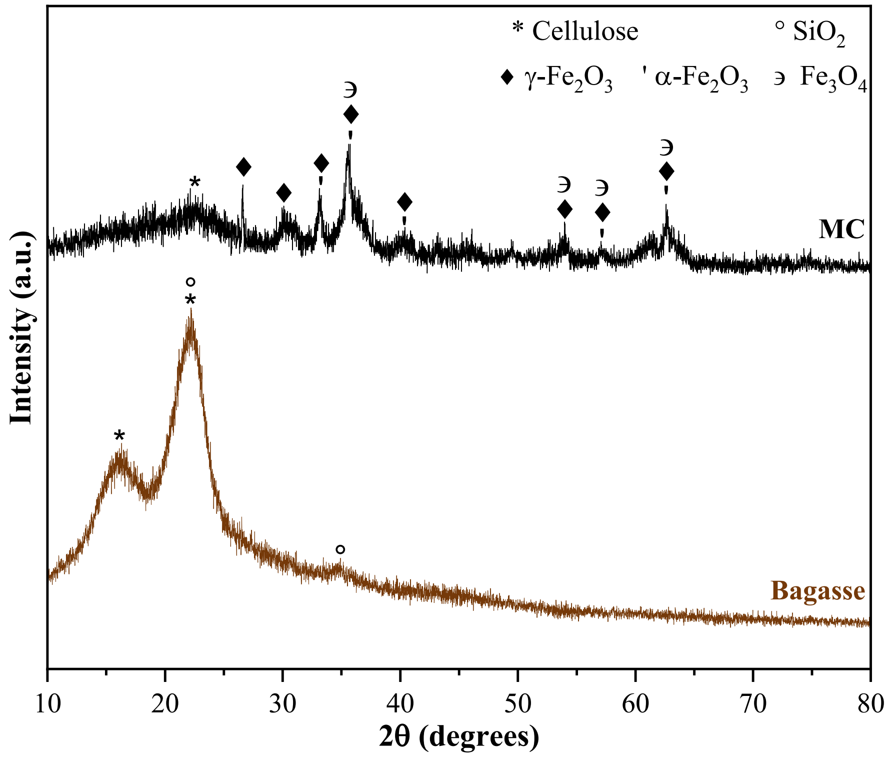

Figure S2. X-ray diffractograms of sugarcane bagasse and MC.

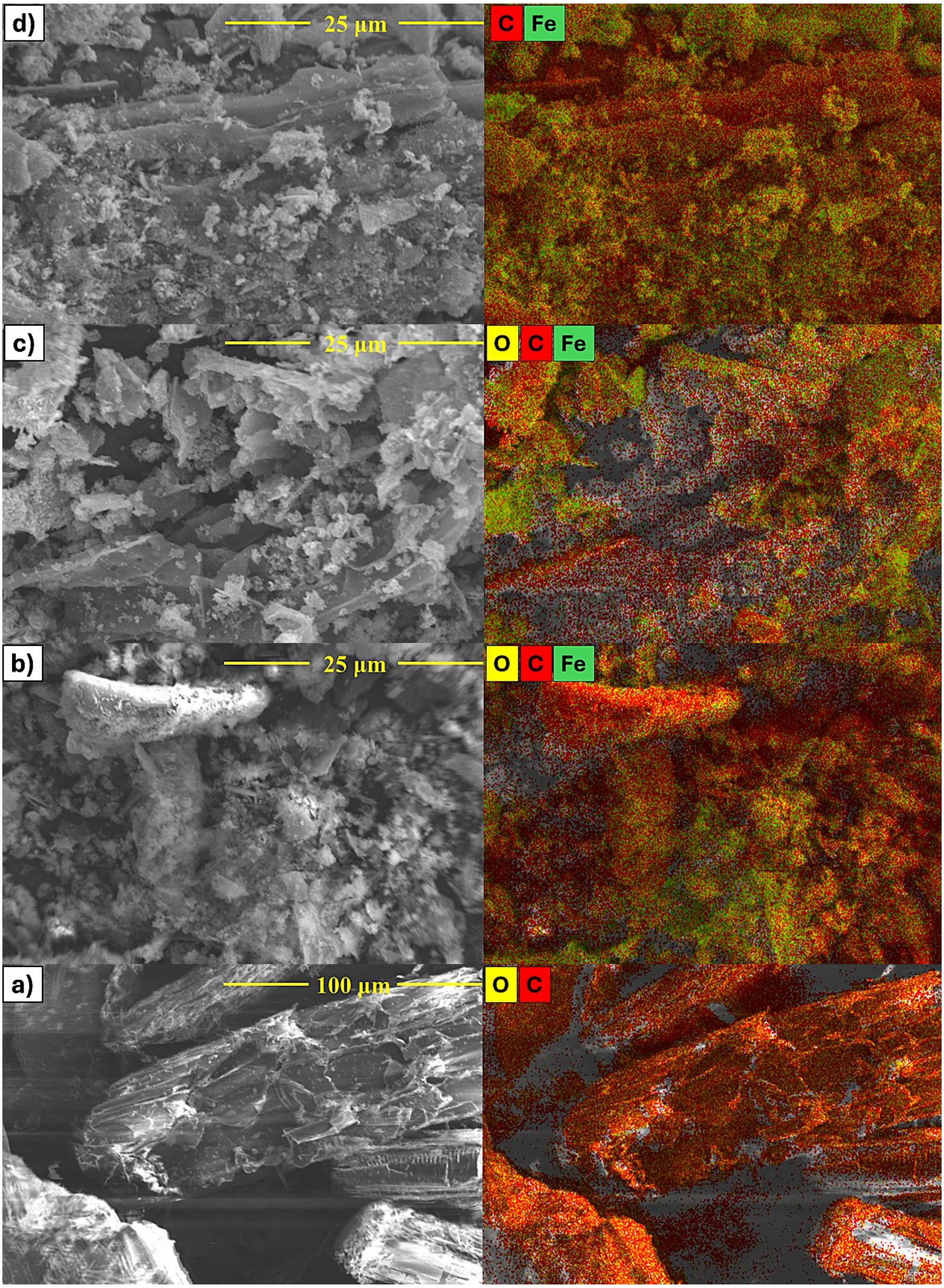

Figure S3. Scanning electron microscopies and elemental mapping for iron, carbon, and oxygen in a) bagasse, b) MC, c) MAC-700-CO_2, and d) MAC-700-N_2.

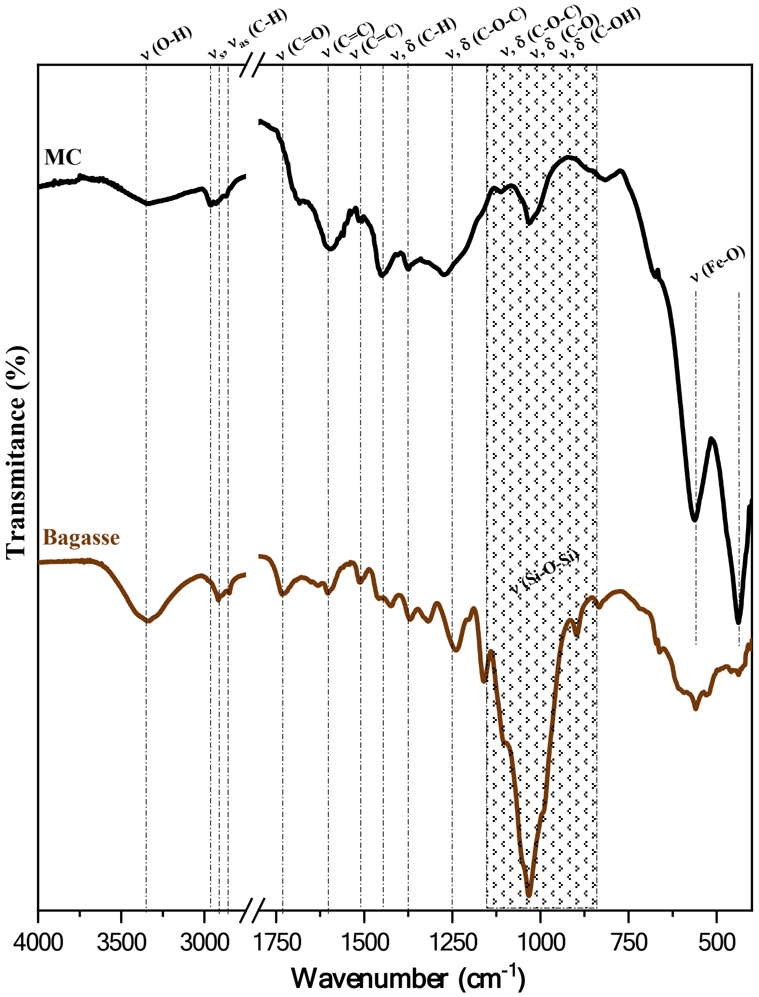

Figure S4. FTIR spectra of the sugarcane bagasse and MC samples.

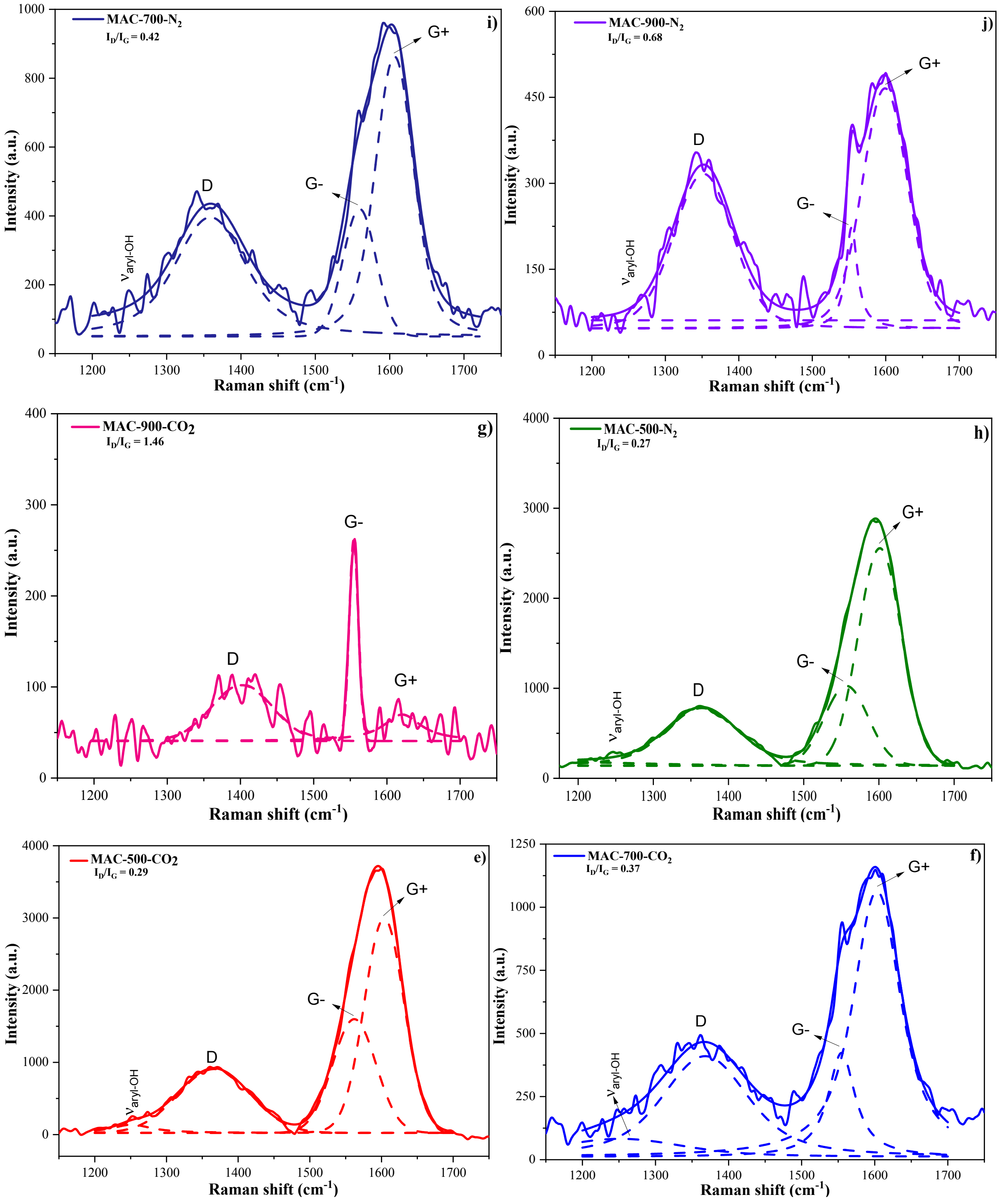
MAC-700-N$_2$
I_D/I_G = 0.42
i)
D
G-
G+
$\nu_{aryl-OH}$
Intensity (a.u.)
Raman shift (cm^{-1})
MAC-900-N$_2$
I_D/I_G = 0.68
j)
D
G-
G+
$\nu_{aryl-OH}$
Intensity (a.u.)
Raman shift (cm^{-1})
MAC-900-CO2
I_D/I_G = 1.46
g)
D
G-
G+
Intensity (a.u.)
Raman shift (cm^{-1})
MAC-500-N$_2$
I_D/I_G = 0.27
h)
D
G-
G+
$\nu_{aryl-OH}$
Intensity (a.u.)
Raman shift (cm^{-1})
MAC-500-CO2
I_D/I_G = 0.29
e)
D
G-
G+
$\nu_{aryl-OH}$
Intensity (a.u.)
Raman shift (cm^{-1})
MAC-700-CO$_2$
I_D/I_G = 0.37
f)
D
G-
G+
$\nu_{aryl-OH}$
Intensity (a.u.)
Raman shift (cm^{-1})

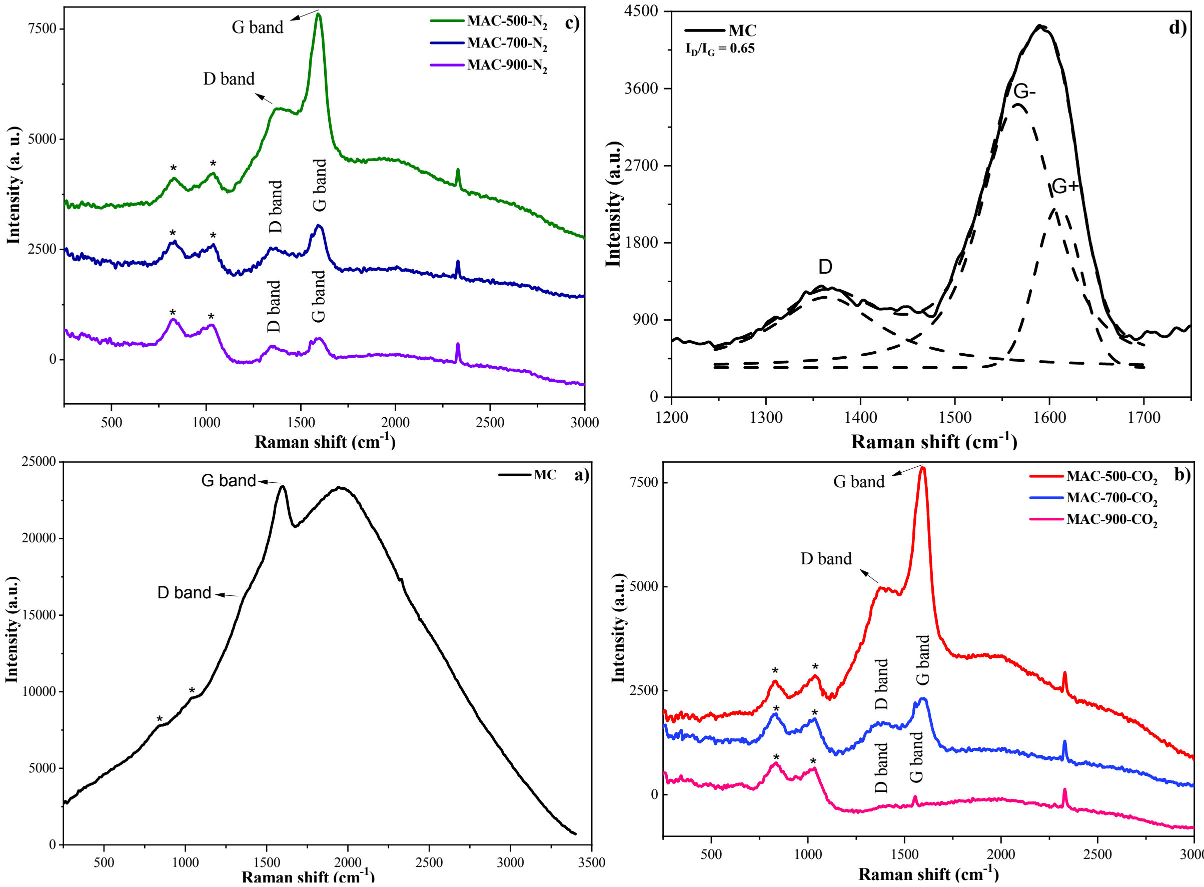

Figure S5. Raman spectra of a) MC, b) MAC-500,700, and 900 °C activated in CO_2 atmosphere; c) MAC-500,700, and 900 °C activated in N_2 atmosphere. Decovoluted spectra of d) MC; e) MAC-500-CO_2; f) MAC-700-CO_2; g) MAC-900-CO_2; h) MAC-500-N_2; i) MAC-700-N_2; j) MAC-900-N_2. *Bands attributed to the substrate where the analysis was carried out.

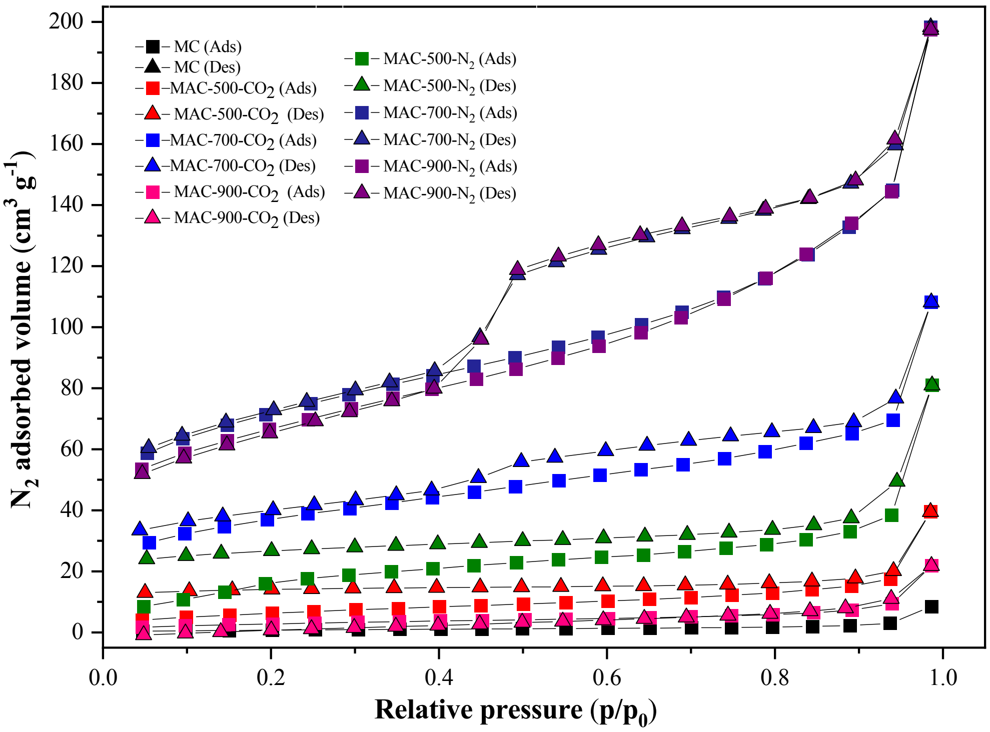

Figure S6. N_2 adsorption-desorption isotherms at -196 °C for MC, MAC-500-CO_2, MAC-700-CO_2, MAC-900-CO_2; MAC-500-N_2, MAC-700-N_2, and MAC-900-N_2.

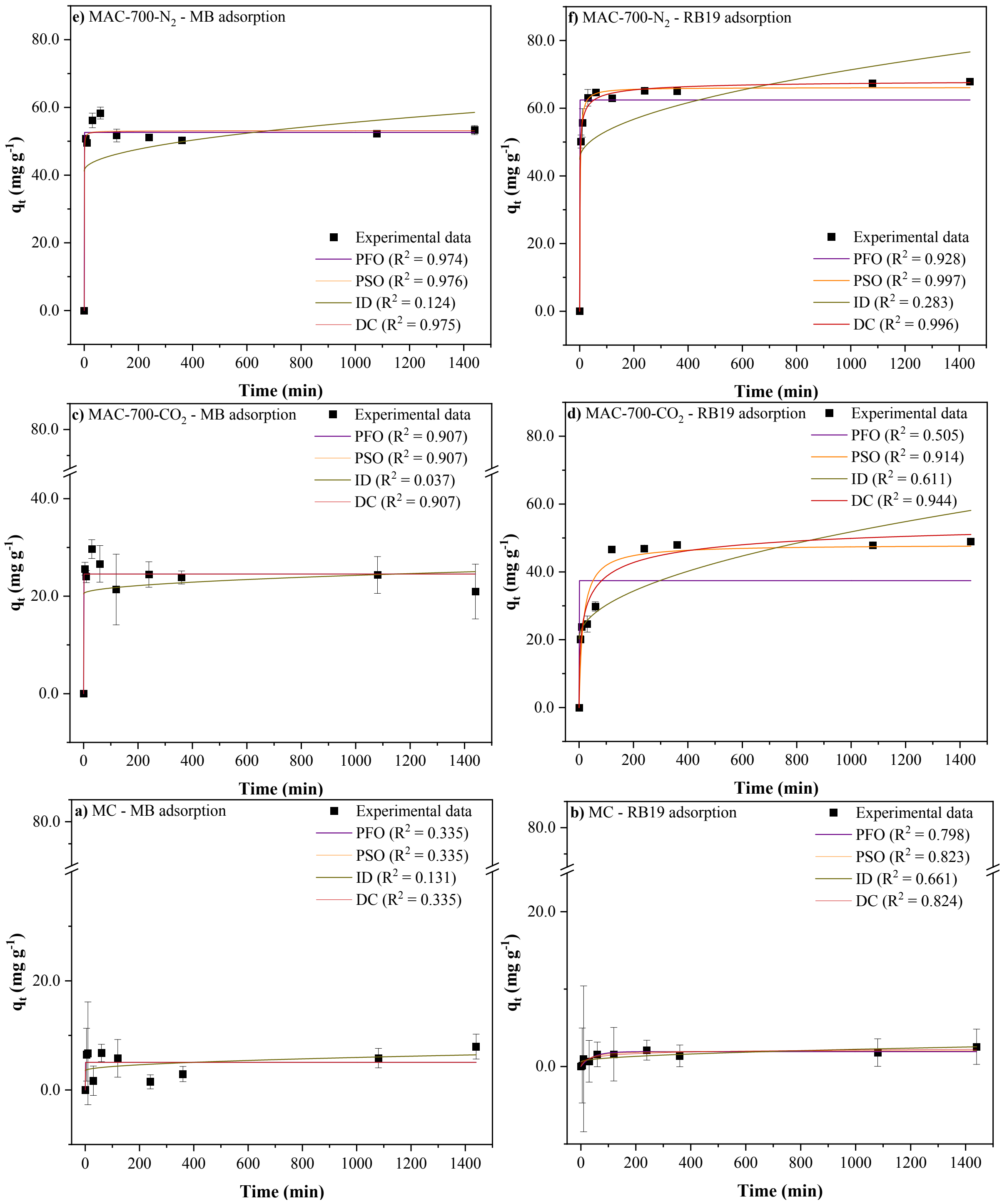

Figure S7. Non-linear fits of the kinetic models of PFO, PSO, ID, and DC in MB a) MC, c) MAC-700-CO_2, and e) MAC-700-N_2; and in RB19 b) MC, d) MAC-700-CO_2, and f) MAC-700-N_2.

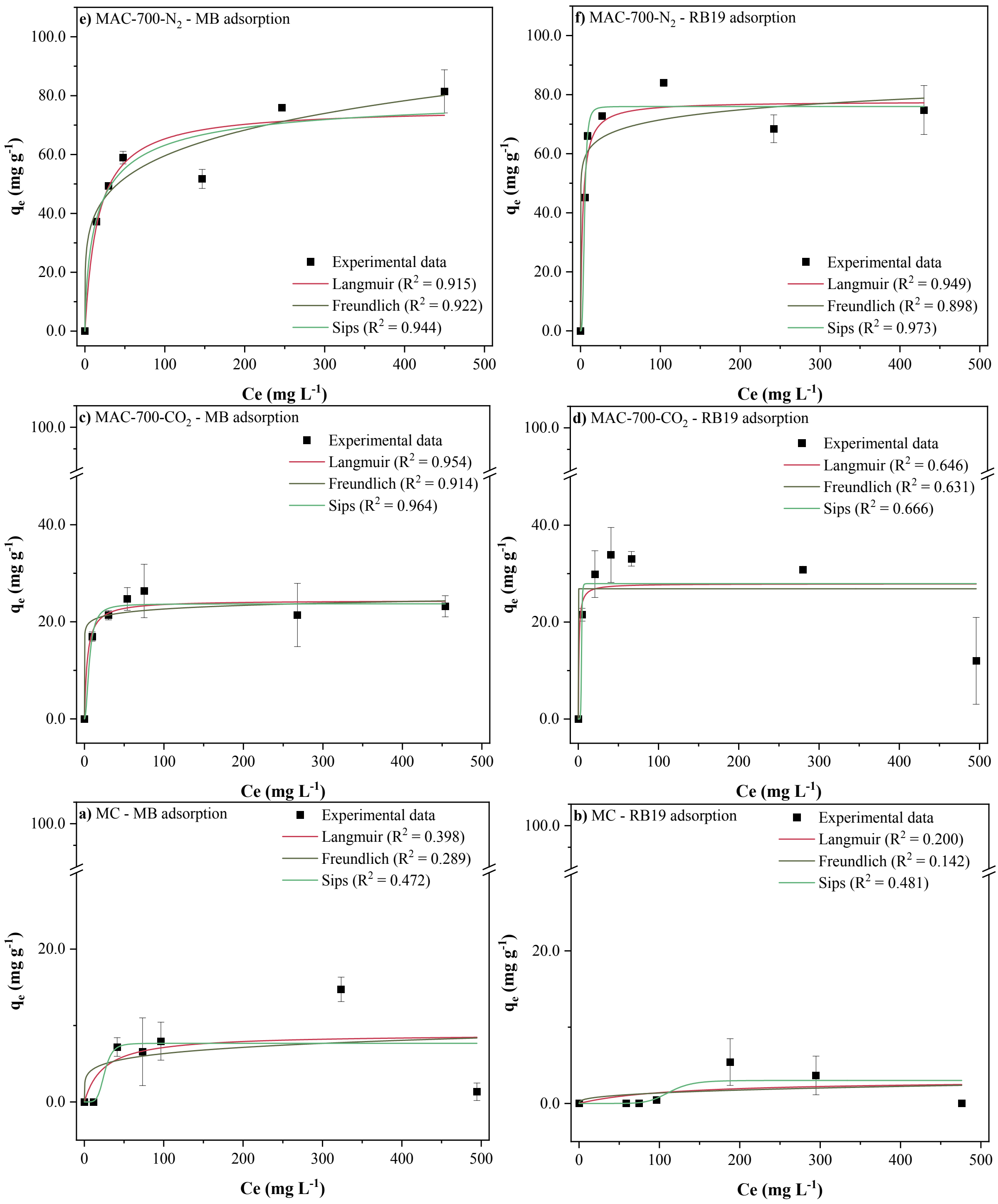

Figure S8. Langmuir, Freundlich, and Sips isothermal non-linear models in MB a) MC, c) MAC-700-CO_2, and e) MAC-700-N_2; and in RB19 b) MC, d) MAC-700-CO_2, and f) MAC-700-N_2.
